# Transcriptional regulation of the carbohydrate utilization network in *Thermotoga maritima*

**DOI:** 10.3389/fmicb.2013.00244

**Published:** 2013-08-23

**Authors:** Dmitry A. Rodionov, Irina A. Rodionova, Xiaoqing Li, Dmitry A. Ravcheev, Yekaterina Tarasova, Vasiliy A. Portnoy, Karsten Zengler, Andrei L. Osterman

**Affiliations:** ^1^Sanford-Burnham Medical Research InstituteLa Jolla, CA, USA; ^2^A. A. Kharkevich Institute for Information Transmission Problems, Russian Academy of SciencesMoscow, Russia; ^3^Department of Bioengineering, University of California San DiegoLa Jolla, CA, USA

**Keywords:** carbohydrate metabolism, transcriptional regulation, regulon, comparative genomics, *Thermotoga*

## Abstract

Hyperthermophilic bacteria from the *Thermotogales* lineage can produce hydrogen by fermenting a wide range of carbohydrates. Previous experimental studies identified a large fraction of genes committed to carbohydrate degradation and utilization in the model bacterium *Thermotoga maritima*. Knowledge of these genes enabled comprehensive reconstruction of biochemical pathways comprising the carbohydrate utilization network. However, transcriptional factors (TFs) and regulatory mechanisms driving this network remained largely unknown. Here, we used an integrated approach based on comparative analysis of genomic and transcriptomic data for the reconstruction of the carbohydrate utilization regulatory networks in 11 *Thermotogales* genomes. We identified DNA-binding motifs and regulons for 19 orthologous TFs in the *Thermotogales*. The inferred regulatory network in *T. maritima* contains 181 genes encoding TFs, sugar catabolic enzymes and ABC-family transporters. In contrast to many previously described bacteria, a transcriptional regulation strategy of *Thermotoga* does not employ global regulatory factors. The reconstructed regulatory network in *T. maritima* was validated by gene expression profiling on a panel of mono- and disaccharides and by *in vitro* DNA-binding assays. The observed upregulation of genes involved in catabolism of pectin, trehalose, cellobiose, arabinose, rhamnose, xylose, glucose, galactose, and ribose showed a strong correlation with the UxaR, TreR, BglR, CelR, AraR, RhaR, XylR, GluR, GalR, and RbsR regulons. Ultimately, this study elucidated the transcriptional regulatory network and mechanisms controlling expression of carbohydrate utilization genes in *T. maritima*. In addition to improving the functional annotations of associated transporters and catabolic enzymes, this research provides novel insights into the evolution of regulatory networks in *Thermotogales*.

## Introduction

Carbohydrates constitute the most abundant single class of organic substances found in nature. Plant cell walls constitute ~70% of the worldwide biomass production by land plants, but only ~2% of this biomass is currently utilized by humans (Pauly and Keegstra, 2010). Carbohydrate composition of plant biomass is highly diverse and differs significantly between plants. A limited number of mono—or disaccharides compose a majority of plant biopolymers. Cellulose, hemicelluloses, and pectins are major polysaccharides of the plant cell wall. Hemicelluloses are characterized by a large diversity of building blocks including pentoses (xylose, arabinose) and hexoses (glucose, mannose, galactose, and rhamnose, and uronic acids). Pectins are composed of galacturonate and rhamnose residues with various branching side chains. Microbial degradation of plant cell wall polysaccharides and their conversion to biofuels is a vital objective for society and requires novel efficient technologies.

Sugar utilization pathways are major feed lines of carbon and energy for central metabolism in a large variety of heterotrophic bacteria. Although these pathways and their transcriptional regulation were extensively studied in model bacteria, projection of this knowledge to diverse bacteria is a major challenge due to chemical diversity of carbohydrates and a matching variability of microbial sugar utilization genes, pathways, and regulons. This variability includes alternative biochemical routes, non-orthologous gene replacements, and functionally heterogeneous families of paralogs. Due to this complexity, genomic annotations of sugar utilization genes derived solely from sequence similarity analysis are often imprecise and incomplete, especially for distantly related bacteria. Metabolic reconstruction based on a combination of various types of genomic context analysis (Yang et al., 2006; Rodionov et al., 2007; Leyn et al., 2012), primarily operons and regulons, allows us to substantially improve the quality of functional annotations and predictions, enabling more accurate metabolic modeling. In this study, we applied an integrative approach to simultaneous genomics-based reconstruction of both metabolic and associated regulatory networks to study carbohydrate utilization networks in a hyperthermophilic marine bacterium from the phylogenetically deep-branching *Thermotogales* group.

**Figure 1:**
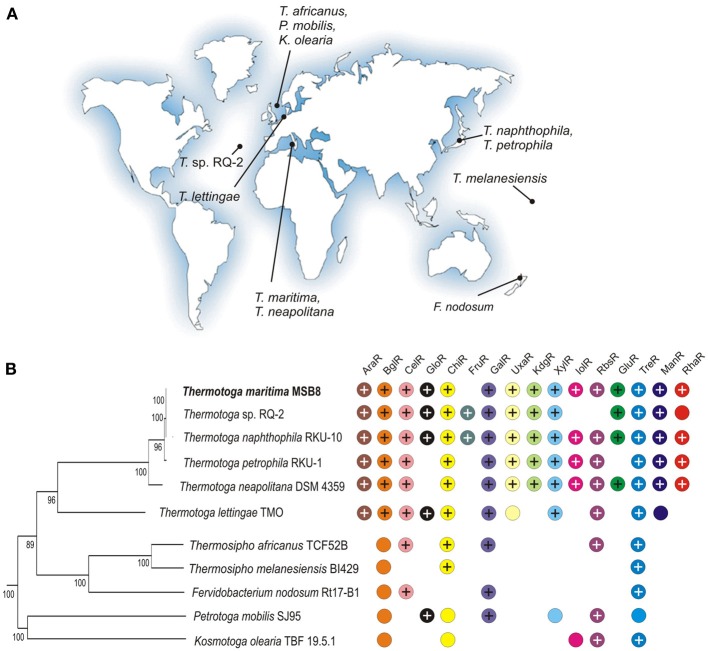
**Properties of 11 *Thermotogales* species analyzed in this study. (A)** The geographic isolation sites for the *Thermotogales* strains. **(B)** Distribution of sugar utilization pathways and cognate transcriptional regulators. The presence of orthologous genes encoding regulators and associated sugar catabolic pathways is shown by “+” and colored circles, respectively. The phylogenetic species tree was constructed using the concatenated alignment of 78 universal bacterial proteins in the MicrobesOnline database (http://www.microbesonline.org/cgi-bin/speciesTree.cgi).

*Thermotoga* spp. are anaerobic fermentative bacteria that are able to grow on various simple and complex carbohydrates including glucose, starch, cellobiose, xylan, and pectin while producing hydrogen, carbon dioxide, and acetate (Chhabra et al., 2002; Kluskens et al., 2003; Conners et al., 2006). *T. maritima* MSB8, a model bacterium in the *Thermotoga* group, was isolated from geothermally heated marine sediments of Volcano Island in Italy (Huber et al., 1986). A closely related bacterium, *T. neapolitana*, was isolated from a submarine thermal vent near Lucrino, Bay of Naples, Italy (Jannasch et al., 1988). Other *Thermotogales* have a broad geographic distribution (see Figure 1) including hydrothermal vents in the Azores [*Themotoga* sp. RQ-2, (Swithers et al., 2011)], a sulfate-reducing bioreactor in Europe [*T. lettingae*, (Balk et al., 2002)], a hot spring in New Zealand [*Fervidobacterium nodosum*, (Patel et al., 1985)], and an offshore oil reservoir in Japan [*T. naphthophila, T. petrophila*, (Takahata et al., 2000)].

The complete genome sequence of *T. maritima* revealed a large proportion of genes (10–15%) involved in carbohydrate metabolism (Nelson et al., 1999). A number of *T. maritima* and *T. neapolitana* enzymes that can degrade β-glucan, hemicelluloses, and pectin have been studied by functional genomics or biochemistry (Conners et al., 2006). A three-dimensional reconstruction of the central metabolic network of *T. maritima* includes 478 enzymes, 120 of which were determined experimentally (Zhang et al., 2009). Still, many metabolic gaps remained in sugar catabolic networks of *T. maritima*. We recently investigated some of those gaps using a combination of bioinformatics and experimental techniques (Rodionova et al., 2012a,b, 2013).

The comprehensive genome-scale metabolic modeling of *T. maritima* also requires an understanding of mechanisms, components, and behavior of the transcriptional regulatory machinery. The integration of genome-scale metabolic and regulatory models significantly improves growth phenotype predictions and allows for the interpretation of systems biology datasets (Faria et al., 2013). Functional genomics approaches were previously used to study global gene expression in response to growth of *T. maritima* on sugars. The transcriptional responses of *T. maritima* to various mono- and polysaccharides identified subsets of genes that are coordinately regulated in response to specific carbohydrates (Chhabra et al., 2003; Nguyen et al., 2004; Conners et al., 2005; Frock et al., 2012). However, the molecular mechanisms underlying these transcriptional responses remain unclear.

To infer sugar-responsive transcriptional regulons in *Thermotoga*, we started from the initial metabolic reconstruction, applying a combination of comparative genomics-based methods of regulatory reconstruction (Rodionov, 2007). Previously, we applied these methods to reconstruct a large number of metabolic regulons in diverse taxonomic groups of bacteria (Rodionov et al., 2011; Leyn et al., 2013; Ravcheev et al., 2013). Integration of metabolic and regulatory reconstructions is particularly efficient for elucidation of carbohydrate utilization networks in a group of taxonomically related bacteria, as recently demonstrated by the comparative genomic study of *Shewanella* spp. (Rodionov et al., 2010). In previous studies, we also combined genomics-based inferences with experimental elucidation to identify and characterize several novel regulons in *Thermotoga*, including Rex controlling the central carbon metabolism and hydrogen production (Ravcheev et al., 2012) and seven ROK-family transcription factors controlling the sugar utilization pathways (Kazanov et al., 2013).

Here, we extended this analysis toward the entire transcriptional network for sugar catabolism in *T. maritima* compared to 10 additional *Thermotogales* species with completely sequenced genomes. The reconstructed networks allowed us to improve gene annotations, refine associated pathways, and identify novel, yet uncharacterized, enzymes and transporters tentatively implicated in the sugar utilization machinery. Some of the critical inferences about newly identified regulons were experimentally verified. A comparison with global transcriptomic data obtained for *T. maritima* grown on different carbohydrates provided additional validation and enrichment of the genomic reconstruction.

## Results

### Genomic reconstruction of sugar catabolic regulons in thermotogales

Initially, we used the subsystems-based bioinformatics workflow (see Methods for details) to identify the repertoire of known and putative genes involved in carbohydrate utilization in *T. maritima* (Table S1 in Supplementary Material). Functional annotation of the identified genes was conducted using the comparative genomic analysis of predicted gene operons and regulons in the *Thermotogales* that was combined with homology searches and metabolic reconstruction. The identified set of ~240 genes encodes the components of at least 19 peripheral sugar utilization pathways as well as the central carbohydrate metabolism (Table 1). In *T. maritima*, the predicted sugar catabolome utilizes 127 enzymes, 22 transport systems (encoded by 86 genes), 18 DNA-binding transcriptional factors, (TFs), and 1 RNA-binding regulator (GlpP).

**Table 1:** **Distribution of the carbohydrate utilization genes and regulators in *T. maritima***.

**Sugar utilization pathway**	**Abbrev.**	**Regulators**	**Transporters**	**Enzymes**	**TOTAL genes**	**TOTAL genes in regulons**
Central carbohydrate metabolism	CCM	–	–	18	18	–
Arabinose, arabinosides	Ara	AraR	AraEFG	6	11	10
β-glucosides	Bgl	BglR	BglEFGKL,	2	9	9
Cellobiose	Cel	CelR	CelEFGKL	7	13	13
Xyloglucan oligosaccharides	Glo	GloR	GloEFGKL	3	9	9
Chitobiose, chitin	Chi	ChiR	ChiEFG	3	7	7
Fructose	Fru	FruR^*^	FruAB^*^	2^*^	7^*^	6^*^
Galactose, galactosides	Gal	GalR	LtpEFGKL, GanEFG	7	16	15
Glycerol	Glp	GlpP	GlpABC, GlpF	2	7	–
Digalacturonate, pectin	Uxa	UxaR	AguEFG	15	19	16
Glucuronate	Kdg	KdgR	*–*	1	3	3
Xylose, xylan	Xyl	XylR	XylEFK, XloEFGKL, XtpEFGKL, XtpN	12	27	27
Inositol	Ino	IolR	InoEFGK	6	11	6
Maltose, maltodextrins	Mal	–	MalEFG	8	15	–
Ribose	Rbs	RbsR	RbsABC	6	12	12
Glucose	Glu	GluR	GluEFK	-	4	4
Trehalose	Tre	TreR	TreEFG	*1*	5	5
Mannose, mannosides	Man	ManR	MtpEFGKL	7	13	11
Rhamnose, rhamnose oligosaccharides	Rha	RhaR	RtpEFGKL	8	14	14
Arabitol, mannitol	Pol	–	–	3	3	–
Hypothetical sugar utilization	HSU	UgtR, UctR, UgpR	UgtEFGK, UctMPQ, UgpEFG	11	24	20
Total number:		19	86	127	241	181

The details of all functional assignments summarized in this table are provided in Table S1. Asterisks indicate that FruR regulator and fructose utilization genes are absent from T. maritima and thus were not counted toward the total numbers.

Next, we identified orthologs for the *T. maritima* sugar catabolic, transport, and regulatory genes in 10 additional *Thermotogales* species with complete genomes (Table S2 in Supplementary Material). Orthologs of the predicted sugar regulators are unevenly distributed among the 11 analyzed genomes (Figure 1). We note that the presence of the orthologous cognate TFs is not ubiquitous for some sugar catabolic pathways. The most conserved TFs in the *Thermotogales* include TreR and GlpR (present in 10 genomes); GalR, GlpP, and UgtR (present in 9 genomes), as well as CelR and ChiR (present in 8 genomes). Three regulators (AraR, BglR, and XylR) were found only in 6 *Thermotoga* genomes, whereas four regulators (UxaR, KdgR, ManR, and UctR) are conserved in 5 closely-related *Thermotoga* spp. Finally, GloR, GluR, InoR, and RhaR have orthologs in a small subset of *Thermotoga* spp.

To infer novel TF regulons, we applied the comparative genomics–based approach implemented in the RegPredict Web server (see Methods). As a result, we revealed DNA motifs and reconstructed regulons for all 18 DNA-binding TFs associated with sugar catabolism in *T. maritima.* In addition, we inferred a regulon for the predicted fructose regulator FruR, which is present in two *Thermotoga* species but not in *T. maritima*. The glycerol regulon operated by the RNA-binding antiterminator protein GlpP was not studied by comparative genomics.

**Figure 2:**
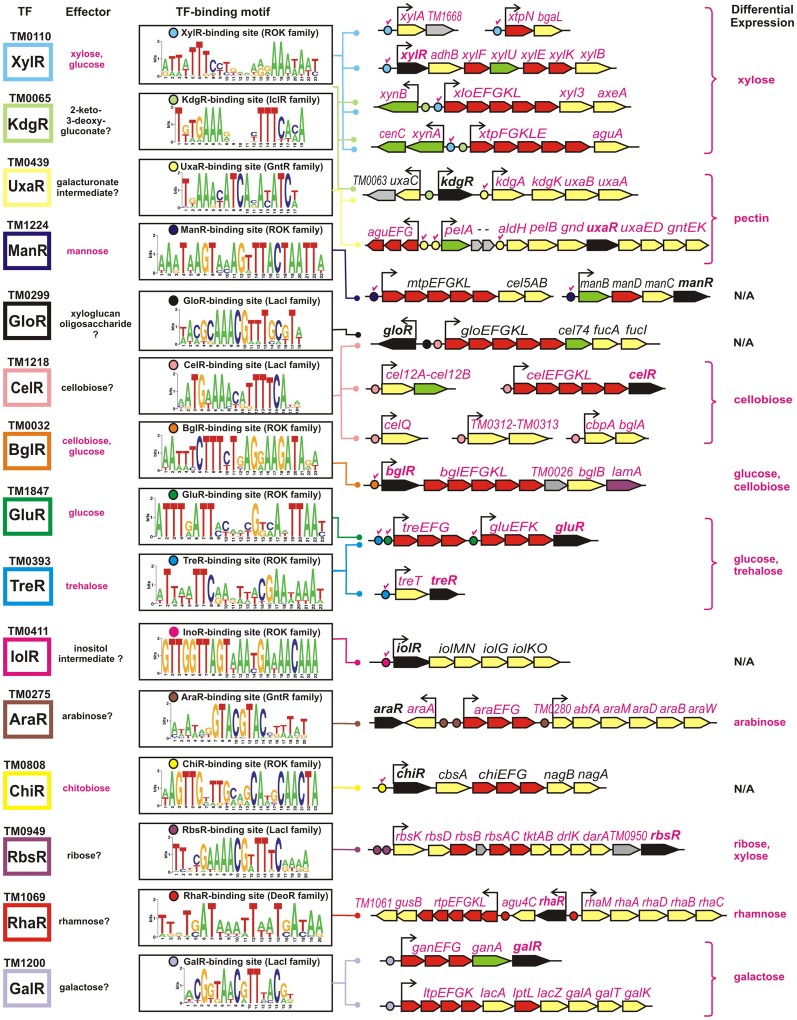
**Genomic context of 15 sugar catabolic regulons in *T. maritima***. Validated and predicted effectors of TFs are listed in red and black, respectively. TF binding sites and downstream regulated genes are shown by circles and arrows, respectively. Validated sites are labeled with red check marks. Sequence logos representing the consensus binding site motifs were built using all candidate sites in the *Thermotogales* genomes. Genes encoding transcriptional regulators and components of sugar transporters are shown in black and red, respectively, whereas the genes encoding the secreted and intracellular sugar catabolic enzymes are in green and yellow, respectively. Hypothetical genes are in gray. Genes that were upregulated on specific sugars (listed in the last column) have their names highlighted in magenta.

**Figure 3:**
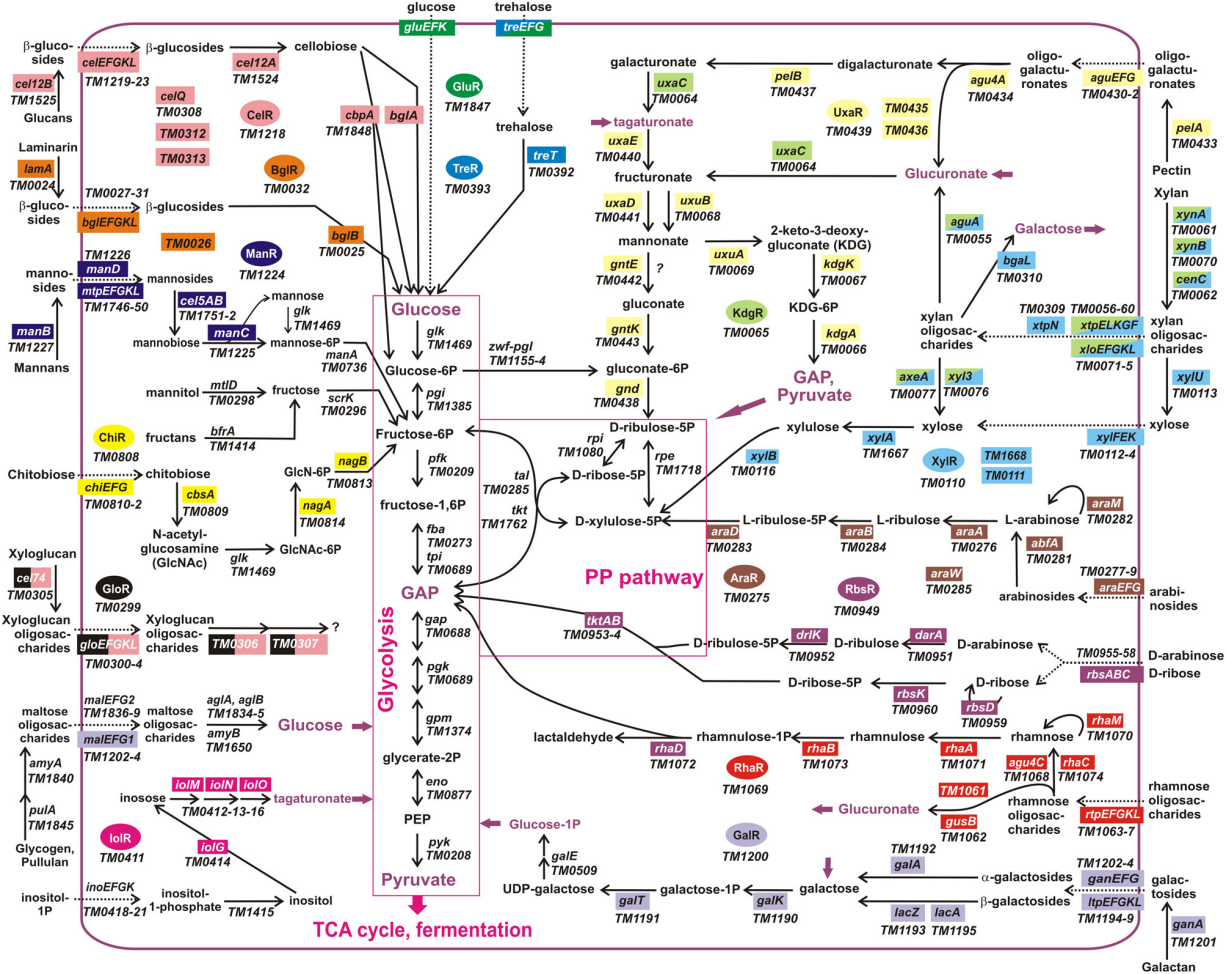
**Integrative reconstruction of sugar utilization pathways and regulons in *T. maritima***. Diagram includes 190 carbohydrate utilization genes encoding catabolic enzymes (solid arrows), transporters (dotted arrows), and transcriptional factors (in ovals of different colors). Genes involved in the reconstructed regulatory network (161 genes regulated by 15 TFs) are highlighted by a matching background color. Three hypothetical sugar utilization pathways and the respective regulons (UgtR, UctR, UgpR) are not shown.

The obtained TF-binding motifs and respective sets of co-regulated operons in *T. maritima* are illustrated in Figure 2. Regulon content for orthologous TFs in other *Thermotogales* genomes is available in the RegPrecise database (Novichkov et al., 2010a) and in Table S2 in Supplementary Material. Metabolic content of 15 functionally annotated regulons is visualized on the integrative *T. maritima* sugar catabolic network (Figure 3). Below, we describe the reconstructed transcriptional regulatory network for sugar catabolism in the *Thermotogales* in more detail.

The xylose-responsive regulon XylR in *T. maritima* contains seven operons organized into five chromosomal gene clusters that encode xylose catabolic enzymes (XylA, XylB), a xylose-specific ABC transporter, a set of hydrolytic enzymes specific for xylan and xylan oligosaccharides, and two xylan-induced ABC transporters. XylR belongs to the ROK (repressor, open reading frame, kinase) protein family. All six predicted XylR-binding sites have been experimentally validated *in vitro* in our previous study of ROK-family regulators (Kazanov et al., 2013). The XylR regulon is well conserved in five *Thermotoga* spp., whereas *T. lettingae* has a reduced size of an orthologous regulon.

The predicted regulator KdgR in *T. maritima* (IclR protein family) is homologous to a repressor of the 2-keto-3-deoxy-gluconate (KDG) metabolism and pectin utilization in enterobacteria (Rodionov et al., 2004). However, in *Thermotoga* spp., the reconstructed KdgR regulon does not include the KDG metabolism genes (*kdgK, kdgA*) but is predicted to control the uronate isomerase *uxaC*, encoding a bifunctional enzyme in the catabolism of glucuronate and galacturonate. Additional candidate KdgR-binding sites were identified in the promoter regions of two XylR-regulated gene clusters, *xynB/xlo* and *xynA/xtp*, that are induced during the growth of *T. maritima* on xylan. Glucuronoxylan is a linear polymer of β-linked xylose residues with many of the xylose units substituted with glucuronate residues. The *xtp* gene cluster encodes α-glucuronidase AguA that can release glucuronate residues from glucuronoxylan. Thus, we propose that the two *Thermotoga* loci under dual regulation by XylR and KdgR are involved in the xylan and glucuronoxylan utilization.

The predicted regulator UxaR (GntR family) was attributed to a conserved 17-nt DNA motif found in multiple copies within the pectin/galacturonate utilization gene loci in five *Thermotoga* genomes (Rodionova et al., 2012a). The UxaR-regulated pectin catabolic locus in *T. maritima* includes pectate lyase *pelA*, digalacturonate ABC transporter *aguEFG*, exo-polygalacturonase *pelB*, and the *gnd, uxaE, uxaD, gntE*, and *gntK* genes required for galacturonate catabolism. Interestingly, the *T. maritima aguEFG* transporter is substituted by other UxaR-regulated transport systems (named *uxaPQM* and *uxaXYZ*) in *T. petrophila, T. naphtophila*, and *T. neapolitana.* A conserved UxaR binding site was identified upstream of the *kdgA-kdgK-uxuB-uxuA* operon encoding enzymes of the glucuronate utilization pathway. These results suggest that the pectin/galacturonate and glucuronate catabolic genes are co-regulated by the transcription factor UxaR in *Thermotoga* spp. The UxaR regulon predicted by *in silico* genomic analysis was experimentally validated in *T. maritima* (see Experimental validation of the inferred regulons in *T. maritima*).

The mannose-responsive regulon ManR (ROK family) includes two operons encoding a set of mannan and mannosides hydrolytic enzymes and a mannan-induced ABC transporter. Both candidate binding sites of ManR and its mannose effector have been previously experimentally tested in *T. maritima* (Kazanov et al., 2013). The ManR-dependent regulation of the *manBDCR* operon is conserved in other *Thermotoga* spp., whereas the ManR-regulated *mtp-cel5* operon is conserved only in the RQ-2 strain.

The predicted cellobiose regulator CelR (LacI family) has an extensive regulon including 22 genes from six genomic loci in *T. maritima*. The conserved part of the CelR regulon (present in five *Thermotoga* genomes) includes glucan and β-glucoside degradation enzymes, cellobiose phosphorylase, and a barley glucan–induced ABC transporter. The latter transporter, termed *celEFGKL*, is also present and regulated by orthologous CelR regulators in three other *Thermotogales* genomes (Figure 1). In *T. maritima*, the reconstructed CelR regulon includes an additional ABC transporter (*gloEFGKL*) encoded within the predicted xyloglucan utilization gene cluster. The LacI-family transcription factor GloR was predicted to function as a local regulator of the *glo* locus. The common intergenic region of *gloR* and *gloE* genes contains distinct operator sites for CelR and GloR regulators. The BglR regulator (ROK family) controls another β-glucosides utilization operon encoding laminarinase, β-glucosidase, and cellobiose- and laminaribiose-specific ABC transporters in *Thermotoga* spp. A BglR-binding site was experimentally validated, and it was found that the BglR regulator from *T. maritima* responds to two effectors, cellobiose and glucose (Kazanov et al., 2013).

**Figure 4:**
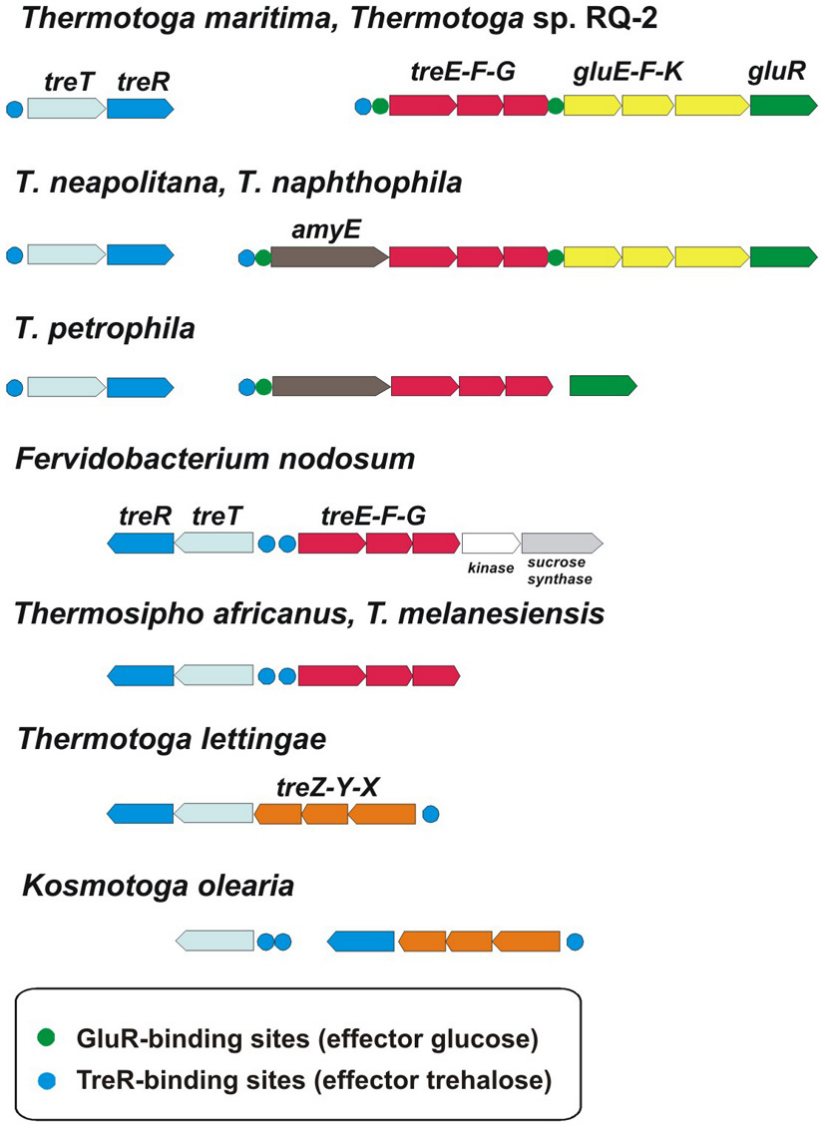
**Genomic context and conservation of trehalose and glucose utilization genes and regulons in *Thermotogales***.

The ROK-family transcription factors GluR and TreR in *T. maritima* control the glucose and trehalose utilization pathways (Kazanov et al., 2013). The trehalose-responsive regulator TreR co-regulates the trehalose ABC transporter *treEFG* and the trehalose synthase-like gene *treT*, whereas GluR controls the glucose ABC transporter *gluEFK* in response to glucose. The glucose regulator has an additional binding site upstream of the *treEFG* operon, which is conserved in five closely related *Thermotoga* genomes (Figure 4). These results suggest that the trehalose transporter is under dual control of both regulators. The TreR regulon is conserved in all studied *Thermotogales* genomes except for *Petrotoga mobilis*.

Two other ROK-family regulators, IolR and ChiR, control the inositol and chitobiose catabolic operons, respectively. The latter operon includes the predicted chitobiose transporter *chiEFG*, the chitobiose hydrolase *cbsA*, and the N-acetylglucosamine (GlcNAc) catabolic genes *nagAB*. Single DNA binding sites of both IolR and ChiR regulators were experimentally confirmed (Kazanov et al., 2013). The specific DNA-binding ability of the ChiR regulator was abolished by a disaccharide chitobiose but not monosaccharide GlcNAc. However, the IolR regulator did not respond to any tested sugar, including glucose, inositol, and inositol phosphate (Kazanov et al., 2013). The IolR regulon is conserved in four *Thermotoga* genomes, whereas the ChiR regulon is present in all studied *Thermotoga* and *Thermosipho* spp. (Figure 1).

The predicted regulator AraR (GntR family) is homologous to the L-arabinose-responsive repressor from *Bacillus subtilis* (Rodionov et al., 2001). The reconstructed AraR regulon in five *Thermotoga* species includes predicted ABC transporters and hydrolytic enzymes for utilization of arabinose oligosaccharides and a set of L-arabinose catabolic enzymes. The predicted ribose regulator RbsR (LacI family) is encoded within the ribose utilization operon. The conserved part of the RbsR regulon in eight *Thermotogales* genomes includes a ribose-specific ABC transporter and a ribokinase. In *T. maritima, T. neapolitana*, and *T. lettingae*, the ribose operon also includes genes encoding two predicted D-arabinose catabolic enzymes and transketolase.

The predicted rhamnose regulator RhaR (DeoR family) was identified within the rhamnose utilization gene cluster in *T. martima* and three other *Thermotoga* spp. The reconstructed RhaR regulon includes a rhamnose-induced ABC transporter, several glycoside hydrolases, and a set of rhamnose catabolic enzymes. The predicted galactose regulator GalR (LacI family) controls the galactan/galactoside utilization pathways in nine *Thermotogales* genomes. In *T. maritima*, the GalR regulon includes two galactose catabolic enzymes, two β-galactosidases, one galactan hydrolase, and two lactose-inducible ABC transporters. The predicted fructose utilization regulon FruR (DeoR family) was found in only two *Thermotoga* spp. (*T. naphthophila* and RQ-2). Finally, three other TF regulons reconstructed in *Thermotoga* genomes control yet uncharacterized sugar catabolic pathways (UctR/TM0326, UgtR/TM1228, and UgpR/TM1856).

### Experimental validation of the inferred regulons in *T. maritima*

**Figure 5:**
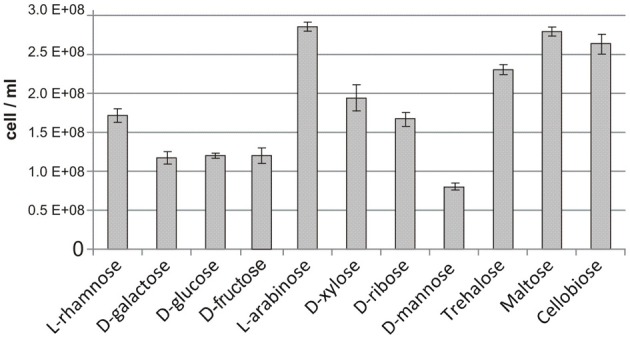
**Maximum cell concentration for *T. maritima* grown on various carbon sources**. Cell density was determined by optical density measured at 600 nm (OD600) after 24 h of cultivation. These values were converted to cell counts using the following equation [cells/mL] = OD600/3.58 × 10^−10^, which was determined from measures of OD600 taken at numerous time points in the growth curve and correlated with flow cytometric cell counts.

We further assessed the reconstructed sugar catabolic regulons by performing genome-wide transcriptional profiling of *T. maritima* cells grown on various mono- and disaccharides. First, the ability of *T. maritima* to grow on 17 different carbon sources was tested in minimal media with a 10-mM concentration of the particular carbohydrate in 500-ml bottles *under anoxic* conditions at 80°C. Growth phenotype testing revealed that *T. maritima* is able to grow on the monosaccharides rhamnose, galactose, glucose, fructose, arabinose, xylose, ribose, and mannose and the disaccharides trehalose, cellobiose, and maltose as a sole carbon and energy source (Figure 5). In contrast, N-acetylglucosamine, gluconate, glucuronate, galacturonate, mannitol, sorbitol, and inositol did not support the growth of *T. maritima* and thus were not used for further transcriptional analysis. The obtained phenotypes are in agreement with the previous studies that have demonstrated growth of *T. maritima* on simple sugars such as glucose, mannose, ribose, arabinose, xylose, and rhamnose (Conners et al., 2005), as well as on the disaccharides maltose and lactose (Nguyen et al., 2004).

Using high-density oligonucleotide tiling arrays, we analyzed changes in transcription levels of *T. maritima* cells grown on different carbon sources (see Methods). The obtained sugar-specific transcriptomes were compared with ribose and other sugars to calculate the differential expression of all genes from regulons reconstructed in *T. maritima* (Table S3 in Supplementary Material). The obtained gene induction patterns for xylose, cellobiose, trehalose, glucose, arabinose, ribose, rhamnose, and galactose correlate with the reconstructed regulons and metabolic effectors for the XylR, BglR, CelR, TreR, GluR, AraR, RbsR, RhaR, and GalR regulators. In addition, the UxaR regulon involving the pectin and galacturonate genes was experimentally validated in *T. maritima* by both the transcriptomic approach and the *in vitro* DNA-binding assays with the recombinant UxaR protein. The genes/operons from these regulons are presented in Figure 2 and discussed below with respect to their differential expression and physiological function in *T. maritima*.

#### XylR regulon

Transcription levels of all 27 genes from the seven operons regulated by the xylose-responsive transcription factor XylR were significantly elevated in *T. maritima* grown on xylose compared to cells grown on ribose. The highest fold changes of gene expression (~10- to 20-fold) were observed for two xylan utilization gene clusters, *xynB<>xloEFGKL-xyl3-axeA* and *xynA-cenC<>xtpFGKLE-aguE*. The xylose catabolic gene cluster *xylR-adhB-xylFUEKB* demonstrated modest upregulation on xylose (~2- to 6-fold), whereas the xylose isomerase *xylA* had approximately 10-fold higher transcript levels. The xylan oligosaccharide utilization operon *xtpN-bgaL* was induced approximately 6-fold. *In vitro*, the XylR regulator responded to 0.02 mM of xylose and also to >0.2 mM of glucose (Kazanov et al., 2013). However, current *in vivo* experiments have not identified any significant induction of the XylR-regulated genes on glucose, suggesting that xylose, rather than glucose, is a physiological inducer of the XylR regulon in *T. maritima*.

#### BglR and CelR regulons

Genes from the BglR-controlled *bglREFGKL-TM0026-bglB-lamA* operon were 4- to 6-fold upregulated during growth on cellobiose (β-glucosyl-1,4-glucose) and 3- to 11-fold induced by glucose. The obtained gene expression profiles for the BglR regulon are corroborated by the previous *in vitro* DNA-binding assays with the BglR repressor that was affected by both cellobiose and glucose, although the glucose disaccharide was a more effective inducer (Kazanov et al., 2013). All 13 genes that belong to five operons from the predicted CelR regulon were highly upregulated during growth on cellobiose but remained uninduced during growth on glucose. The *cbpA* and *celQ* genes showed the highest induction by cellobiose (more than 10-fold), whereas the responses of the *celEFGKLR, cel12AB*, and *TM0312-13* operons to cellobiose were more moderate (5- to 9-fold). In contrast, the putative xyloglucan oligosaccharide utilization gene cluster *gloR<>gloEFGKL-cel74-fucAI*, which was predicted as a part of the CelR regulon, was uninduced on cellobiose or other sugars tested. The latter fact can be explained by transcriptional repression of the *xlo* gene locus by another LacI-family predicted regulator, GloR, that has a yet-unknown sugar effector. Based on these results, we concluded that cellobiose is a physiological effector that induces the CelR regulon in *T. maritima*.

#### TreR and GluR regulons

**Figure 6:**
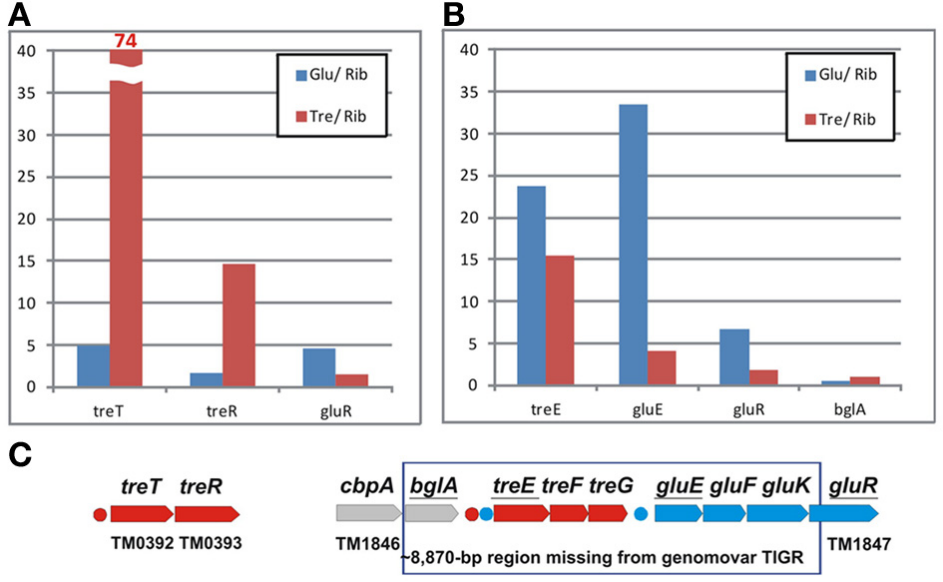
**Differential expression of trehalose and glucose utilization genes in *T. maritima*. (A)** Gene expression fold changes for the *treTR* and *gluR* genes for the cells grown on glucose (Glu) and trehalose (Tre) vs. ribose (Rib), as determined by whole-genome microarrays. **(B)** Gene expression fold changes for the *treE, gluE*, and *gluR* genes as determined by RT-PCR. The *bglA* gene was used as a negative control. Normalized transcript levels with respect to the TM0688 (*gap*) gene are provided in Figure S1 in Supplementary Material. The mean of biological duplicate measurements was used. **(C)** Genomic clusters of genes involved in the trehalose (red) and glucose (blue) utilizations.

The TreR-regulated *treTR* operon encoding a putative trehalose utilization enzyme and the trehalose-responsive repressor was highly upregulated in *T. maritima* cells grown on trehalose (Figure 6A). Although the DNA microarray hybridization confirmed the *in vivo* induction of *treTR* by trehalose, similar analysis of the *treEFG* and *gluEFK* genes was not possible due to the absence of the respective oligonucleotide probes. The *T. maritima* MSB8 isolate that was originally sequenced in 1999 [TIGR genomovar, (Nelson et al., 1999)] and that was used for microarray gene probe design lacks the 8870-bp DNA region between the genes *TM1846* (*cbpA*) and *TM1847* (*gluR*). This DNA region is present in two other *T. maritima* MSB8 isolates that have recently been resequenced [genomovars DSM 3109 (Boucher and Noll, 2011) and ATCC (Latif et al., 2013)]. The new region includes seven genes including *bglA*, a member of the CelR-regulated *cbpA-bglA* operon, and the *treEFG* and *gluEFK* genes that are regulated by TreR and GluR (Kazanov et al., 2013) (Figure 6C). To validate sugar-specific induction of these two operons *in vivo*, we performed real-time reverse transcription PCR (RT-PCR) with probes designed for *treE, gluE, gluR*, and *bglA* (used as a negative control) (Figure 6B). All three genes tested had demonstrated elevated expression levels in *T. maritima* cells grown on either glucose or trehalose compared to the cells grown on ribose. The highest fold changes in gene expression were observed for *treE* and *gluE* in the glucose-grown cells, whereas in the trehalose-grown cells only the *treE* gene was highly upregulated. These results confirm the dual control of the trehalose transporter *treEFG* by both TreR and GluR regulators in response to trehalose and glucose, respectively. Also, the RT-PCR results confirm that the glucose transporter *gluEFGR* operon is induced by glucose using the glucose-responsive repressor GluR.

#### AraR regulon

All genes from the predicted AraR regulon that are organized in three transcriptional units were upregulated in *T. martima* cells grown on arabinose compared to cells grown on ribose. The *TM0280-abfA* and *araA* genes demonstrated the highest induction levels (61- to 161-fold), the arabinoside transporter genes *araEFG* were modestly induced (9- to 16-fold), whereas expression of the arabinose catabolic genes *araMDBW* was increased only 2- to 6-fold. Large differences in the induction levels between the *TM0280-abfA* and *araMDBW* genes can be attributed to the putative transcriptional terminator upstream of *araM* (Latif et al., 2013). These results suggest that arabinose is a physiological inducer for the AraR regulon. This hypothesis is supported by the fact that AraR orthologs in *Clostridium acetobutylicum* and *B. subtilis* function as arabinose-responsive repressors (Zhang et al., 2012).

#### RbsR regulon

Transcription of all genes from the ribose utilization operon was highly upregulated in *T. maritima* cells grown on ribose compared to cells grown on glucose (14- to 92-fold). Unexpectedly, the ribose operon genes were almost equally highly induced in cells grown on xylose. The ribose operon is predicted to be controlled by the LacI-family regulator RbsR, whose molecular effector is not yet experimentally determined. The unusual induction patterns for ribose utilization genes may be explained by the dual specificity of the RbsR repressor to ribose and xylose. Alternatively, since the xylose utilization feeds the pentose-phosphate pathway, it is possible that ribose 5-phosphate, an intermediate of this pathway, serves as a physiological effector of the RbsR regulon in *T. maritima*.

#### RhaR regulon

The rhamnose oligosaccharide utilization gene cluster in *T. maritima* includes 14 genes organized in three transcriptional units that are predicted to be regulated by RhaR. Transcription of all three RhaR-regulated operons was highly elevated in *T. maritima* cells grown on rhamnose compared to cells grown on ribose. The rhamnose catabolic genes from the *rhaMADBC* operon were induced between 27- and 37-fold, the *rhaR-agu4C* operon showed ~12-fold induction, whereas the *rtpEFGKL-gusB-TM1061* operon genes demonstrated the gradual decrease in their induction level from 60-fold for the first gene to ~5-fold for the latter gene. These expression results suggest that the RhaR regulator in *T. maritima* responds to the rhamnose monosaccharide.

#### GalR regulon

The predicted galactoside utilization regulon GalR in *T. maritima* includes 15 genes organized into two operons, both of which were upregulated during growth on galactose. Genes encoding putative transporters for galactose oligosaccharide and galactosides (*ganEFG* and *ltpEFGKL*), as well as two β-galactosidases (*lacA* and *ganA*), demonstrated the highest levels of induction on galactose compared to ribose (8- to 20-fold). The galactose catabolic genes (*galT* and *galK*) and the putative galactose repressor gene *galR* were modestly induced by galactose (~4-fold). These results are in agreement with the previous microarray expression profiling of *T. maritima* that have demonstrated induction of all above-mentioned GalR regulon genes in cells grown on lactose but not on maltose (Nguyen et al., 2004).

#### UxaR regulon

**Figure 7:**
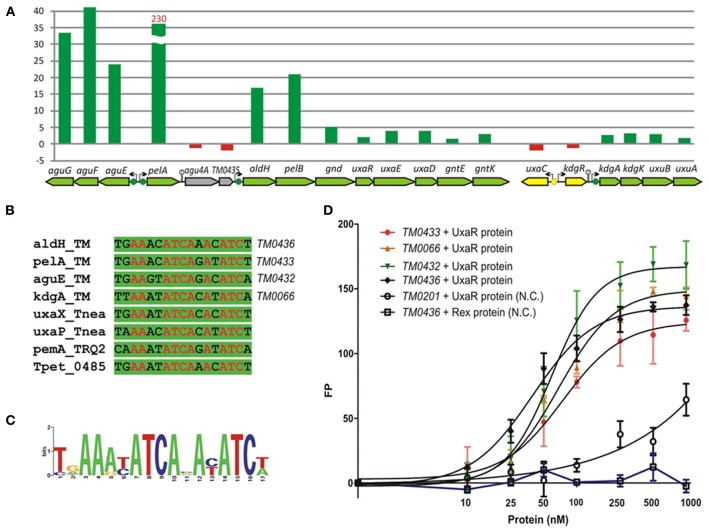
**Experimental validation of UxaR regulon in *T. maritima*. (A)** Non-redundant set of UxaR binding sites identified in *T. maritima* (TM), *T. neapolitana* (Tnea), *Thermotoga* sp. RQ-2 (TRQ2), and *T. petrophila* (Tpet). **(B)** Sequence logo for UxaR binding sites. **(C)** Fluorescence polarization binding assay of UxaR with target DNA operators in *T. maritima*. Increasing protein concentrations were mixed with 27-bp fluorescence-labeled DNA fragments of gene regions containing UxaR-binding sites. As a negative control (N.C.), the *T. maritima* regulator Rex (TM0169) and DNA fragment of the *TM0201* gene containing the Rex-binding site were used. **(D)** Gene expression fold change for *T. maritima* grown on pectin vs. ribose.

The GntR-type transcriptional regulator UxaR was predicted to bind a conserved DNA motif in regulatory regions of four pectin and galacturonate utilization operons in *T. maritima* (Figures 7A,B). The UxaR regulon includes the extracytoplasmic pectate lyase PelA, the digalacturonate-specific transporter AguEFG, the cytoplasmic polygalacturonase PelB, and the extensive set of galacturonate catabolic enzymes (Rodionova et al., 2012a). We performed experimental validation of the predicted UxaR regulon by both *in vitro* and *in vivo* approaches. To assess specific binding of the purified recombinant UxaR protein to the predicted UxaR operators in *T. maritima* we used a fluorescence polarization assay (Figure 7C). The results show that UxaR specifically binds to the synthetic 27-nt DNA fragments containing UxaR binding sites. All tested DNA fragments demonstrated the concentration-dependent increase of fluorescence polarization, confirming specific interaction between the regulator and DNA fragments. The apparent *K*_*d*_ values for the UxaR protein interacting with the tested DNA fragments were in the range of 40–80 nM. We also tested the influence of potential sugar effectors on protein-DNA interaction, however, the addition of digalacturonate, galacturonate, glucuronate, or hexuronate catabolic pathway intermediates had no effect on the complex formation (data not shown).

Galacturonate does not support the growth of *T. maritima* when used as a single carbon source (data not shown). Thus, we assessed gene expression in cells grown on pectin, a galacturonate-containing polysaccharide, which can support the growth of *T. maritima* (Kluskens et al., 2003). The expression of ~50 sugar catabolic genes was significantly (>2-fold) upregulated in cells grown on pectin compared to cells grown on ribose (Table S3 in Supplementary Material). In particular, the expression of 6 genes from the UxaR regulon (*aguEFG, pelA*, and *aldH-pelB*) showed more than 17-fold upregulation (Figure 7D). The extracellular pectate lyase gene *pelA* was expressed 230-fold higher on pectin when compared to ribose. Most of the remaining UxaR-regulated genes from the *aldH-pelB-gnd-uxaRED-gntEK* and *kdgAK-uxuBA* operons showed a 2- to 5-fold upregulation on pectin. The *agu4A-TM0435* genes located immediately downstream of *pelA* did not show any upregulation on pectin, suggesting that they are not co-transcribed with *pelA*. Accordingly, we found a putative rho-independent transcriptional terminator downstream of *pelA*. In addition to the induction of pectin/galacturonate catabolic genes, three gene clusters encoding the beta-glucoside (*TM0024-32*), arabinose (*TM0276-284*), and galactoside (*TM1190-1204*) utilization pathways were significantly upregulated in *T. maritima* cells grown on pectin. These genes clusters do not belong to the UxaR regulon but were predicted to be regulated by BglR, AraR, and GalR, respectively. Induction of these three regulons by pectin may be explained by the presence of glucose, arabinose, and galactose residues in this polysaccharide.

## Discussion

Transcriptional regulation is a highly variable component of carbohydrate utilization networks. Although our current knowledge of sugar catabolic regulons in model bacteria such as *Escherichia coli* and *Bacillus subtilis* is nearly comprehensive, the projection of this knowledge to the sequenced genomes of bacteria from distant taxonomic groups is still a challenge. The major difficulties for bioinformatics-based propagation of TF regulons include duplications and losses of TFs and their binding sites, rapid diversification of specificities of TFs toward DNA sites and sugar effectors, non-orthologous replacements of entire regulatory systems, and frequent horizontal transfers. Comparative genomic analysis of specific sugar utilization pathways has led to substantial progress in the identification and reconstruction of their cognate TF regulons in diverse bacterial lineages (Yang et al., 2006; Rodionov et al., 2010; Leyn et al., 2012). In this study, we used comparative genomics to reconstruct novel TF regulons for sugar catabolism in hyperthermophilic bacteria from the deep-branching lineage *Thermotogales* (Figure 1). In *T. maritima*, a model bacterium in this lineage, the identified sugar catabolic regulatory network includes 18 TFs, 40 TF binding sites, and 163 target genes comprising 15 known and three hypothetical metabolic pathways (Table 1; Figures 2, 3). All studied sugar-responsive TFs in *T. maritima* are encoded within their cognate regulated gene loci, and thus are subject to autoregulation.

All sugar utilization regulons reconstructed in *Thermotogales* are controlled by TFs that are homologous to known sugar-related regulators from six families: DeoR, GntR, IclR, LacI, ROK, and RpiR. For most of these TFs, phylogenetic and genome context analysis of distant homologs did not reveal their potential functional orthologs outside the *Thermotogales* lineage. These genomic observations suggest that the analyzed sugar metabolic regulators have evolved and functionally specialized after the separation of the *Thermotogales*. Our previous phylogenetic analysis suggests that the expansion of the ROK family represented by seven paralogs in *T. maritima* was likely due to massive duplications and subsequent functional diversification of regulators during the evolution of *Thermotogales* (Kazanov et al., 2013). The DeoR-family regulator FruR controlling the fructose utilization operon in two closely related *Thermotoga* spp. represents an exceptional case of relatively recent horizontal gene transfer from thermophilic Clostridia. All genes from the fructose utilization operon in *Thermotoga* spp., including *fruR*, are highly similar to the orthologous genes in *Caldicellulosiruptor* spp. (70–80% identity). We propose that the entire fructose utilization operon was laterally transferred between these two lineages that likely share the same ecological niche.

The observed variations in the distribution of sugar utilization regulons among the *Thermotogales* are also remarkable (Figure 1). Only six regulons are present in at least two different genera (CelR, ChiR, GalR, GloR, RbsR, and TreR), whereas the remaining regulons are present only in the *Thermotoga* genus. Moreover, most of the latter regulons are restricted to the group of five closely related *Thermotoga* spp, and only three regulons have orthologs in a more distant genome of *T. lettingae*. Conservation of all *Thermotoga*-specific regulons except FruR suggests their likely emergence in the common ancestor of this genus. We observed several cases of species-specific regulon loss in which an entire regulon (including all operons from a regulated pathway) is missing only in a single *Thermotoga* spp., including the inositol and ribose utilization regulons in the RQ-2 strain and the glucose utilization regulon GluR in *T. petrophila*. In another case, the RhaR regulator was lost in a single strain of *Thermotoga* sp. RQ-2, however, the rhamnose utilization operons are still retained in the genome. Many other cases of the absence of orthologous regulators for sugar catabolic pathways in the genomes of more distant *Thermotogales* can be explained by their control by non-orthologous TFs that have yet to be characterized (Figure 1).

Overall, the reconstructed carbohydrate utilization regulatory network in *T. maritima* contains 18 local TF regulons (FruR is absent from *T. maritima*), each controlling between one and seven operons. In contrast to other model bacteria (e.g., *E. coli* and *B. subtilis*) that employ various transcriptional mechanisms for global catabolite repression of sugar metabolism, our genomic-based analysis did not reveal any global regulons for sugar utilization genes in *Thermotoga*. This observation correlates with the genome-wide transciptome data for *T. maritima* obtained in this and previous studies (Chhabra et al., 2003; Frock et al., 2012). With the exception of a fructose-specific system in two *Thermotoga* strains, the *Thermotogales* genomes lack orthologs of sugar phosphotranferase (PTS) systems that are essential components of global carbon catabolic repression mechanisms in both Gram-negative and Gram-positive bacteria (Deutscher, 2008). Thus, bacteria from the Thermotogae phylum appear to use different regulatory strategies for sugar utilization.

Another remarkable feature of sugar catabolic networks in *Thermotoga* is the existence of multiple interconnections between distinct sugar regulons. Two xylan catabolic gene loci, *xtp* and *xlo*, were found to be controlled by the xylose-responsive regulator XylR and the predicted glucuronate utilization regulator KdgR in *T. maritima* (Figure 2) and in other *Thermotoga* genomes (Table S2 in Supplementary Material). The first gene locus encodes a secreted endoxylanase, a putative xylan oligosaccharide ABC transporter, and a cytoplasmic glucuronidase, which might be involved in the cleavage of glucuronate residues from a xylan-derived oligosaccharide. The second gene locus encodes another secreted endoxylanase, a xylose oligosaccharide ABC transporter, and a cytoplasmic xylosidase and is presumably involved in the utilization of xylose-containing oligosaccharides. From transcriptomic data in *T. maritima*, both XylR/KdgR-regulated gene loci are upregulated during growth on xylose (Tables S3 in Supplementary Material) and xylan (Conners et al., 2005). Another gene locus, *glo*, encoding hypothetical glucose oligosaccharide utilization genes, is predicted to be controlled by both the locally encoded regulator GloR and the distal regulator CelR, which also controls five other operons involved in cellobiose and glucan utilization (Figure 2). Lastly, the trehalose transporter *treEFG* in five *Thermotoga* spp. is controlled by the trehalose- and glucose-responsive regulators TreR and GluR, respectively (Figure 4). We confirmed by RT-PCR that the *treE* gene in *T. maritima* is upregulated on glucose and trehalose (Figure 7). The observed partial overlaps between distinct local regulons in *Thermotoga* point to the existence of coordinated regulatory responses to particular types of complex polysaccharides and disaccharides.

We assessed the reconstructed sugar regulatory network in *T. maritima* by microarray gene expression profiling of cells grown on a variety of mono- and disaccharides and pectin as a single carbon source. The obtained gene induction patterns demonstrate overall consistency with individual regulons reconstructed by genomic analysis. In addition, the previously determined effectors of the ROK-family regulators XylR, TreR, GluR, and BglR (Kazanov et al., 2013) were found to be in good agreement with the *in vivo* gene expression results for xylose, trehalose, glucose, and cellobiose, respectively (Figure 2). In addition, the reconstructed regulons correlated with previous microarray expression studies in *T. maritima* (Chhabra et al., 2003; Nguyen et al., 2004; Conners et al., 2005). For instance, the predicted galactoside utilization regulon GalR was induced by galactose in our study and was previously shown to be upregulated on lactose, a galactose-glucose disaccharide (Nguyen et al., 2004). The predicted cellobiose and glucan utilization regulon CelR was induced by cellobiose in our study and was previously shown to be upregulated by barley and glucomannan (Conners et al., 2005).

The reconstructed regulatory network allowed us to suggest and/or refine specific functional assignments for sugar-specific ABC transporters that constitute the most abundant class of uptake transporters in sugar utilization pathways of *Thermotogales*. In *T. maritima*, the previously uncharacterized transporter ChiEFG was tentatively assigned chitobiose specificity based on the chitobiose-responsive regulon ChiR (Figure 3). Similarly, the characterized GluR and TreR regulons and their respective effectors allowed us to propose glucose and trehalose specificities for the GluEFK and TreEFG transporters, respectively, and these functional assignments were recently experimentally validated (Boucher and Noll, 2011). Finally, the reconstructed regulons of other *Thermotoga* species allowed us to predict sugar specificities for multiple novel ABC transporters that are non-orthologous to transport systems from *T. maritima* (Table S1 in Supplementary Material). Interestingly, despite of the absence of FruR regulon, *T. maritima* demonstrates some growth on fructose as a sole carbon source (Figure 5). This observation correlates with the presence of functional fructokinase TM0296 (Rodionova et al., 2012b), however, the fructose uptake transporter remains unknown in *T. maritima*.

In summary, the comparative genomics-based regulon and pathway reconstruction combined with some experimental data allowed us to identify the integrated metabolic and regulatory network of sugar utilization in *T. maritima* and revealed its substantial diversity between different *Thermotogales* species. Our results revealed a high level of consistency between the *in silico*–predicted TF regulons, the *in vitro*–determined TF effector specificities, and the *in vivo*–measured gene expression changes. The described integrative genomics–based approach for regulon analysis may be applied to other yet uncharacterized taxonomic groups of microorganisms for which multiple closely related genomes are available.

## Materials and methods

### Functional gene annotation and metabolic reconstruction

To map carbohydrate utilization genes in the *T. maritima* genome, we used the bioinformatic workflow that was previously applied for the analysis of *Shewanella* genomes (Rodionov et al., 2010). The workflow is based on a collection of manually curated subsystems in the SEED genomic database capturing a substantial fraction of known sugar utilization pathways projected across microbial genomes (Overbeek et al., 2005). Using a compilation of ~500 groups of isofunctional homologs from various sugar utilization subsystems in SEED, we performed homology-based scanning of the *T. maritima* genome. Based on genome context analysis of the identified gene candidates, we finally collected ~240 genes potentially involved in carbohydrate utilization in *T. maritima*. We also collected experimental knowledge and literature references for ~130 proteins from the collection including 62 sugar catabolic enzymes, 12 binding components of ABC transporters, and 6 regulators with established cognate metabolites (substrates, ligands, or effectors). For the remaining ~50 *T. maritima* proteins, the previously reported gene expression profiles revealed their upregulation during the growth on specific poly- or monosaccharides used as a single carbon source (Nguyen et al., 2004; Conners et al., 2005; Frock et al., 2012). The obtained functional gene annotations are captured in the SEED subsystem available online at http://pubseed.theseed.org/SubsysEditor.cgi?page=ShowSubsystemandsubsystem=Sugarutilizationin Thermotogales and summarized in Table S1 in Supplementary Material.

### Genomic reconstruction of regulons

Putative TF regulons were reconstructed using the established comparative genomic approach [reviewed in (Rodionov, 2007)] implemented in the RegPredict Web server (http://regpredict.lbl.gov) (Novichkov et al., 2010b). We started from the genomic identification of reference sets of genomes that encodes orthologs of each TF and their genome context analysis. Initial training sets of potentially co-regulated genes were collected based on the gene neighborhood analysis of TF orthologs in the MicrobesOnLine database (http://microbesonline.org/) (Dehal et al., 2010). For *de novo* identification of a candidate TF-binding motif in the training set of potential upstream regions, we used the “Discover Profile” procedure implemented in RegPredict. Each identified DNA motif was used to scan the *Thermotogales* genomes to predict novel regulon members. Scores of candidate sites were calculated as the sum of positional nucleotide weights. The score threshold was defined as the lowest score observed in the training set. The conserved regulatory interactions with high-scored binding sites that involve target genes involved in carbohydrate metabolism were included in the reconstructed regulons. Candidate sites associated with new members of regulons were added to the training set, and the respective group profile was rebuilt to improve search accuracy. Sequence logos for the derived group-specific DNA binding motifs were drawn using the WebLogo package (Crooks et al., 2004). The details of all reconstructed regulons are captured and displayed in RegPrecise, a specialized database of bacterial regulons (http://regprecise.lbl.gov) (Novichkov et al., 2010a), as a part of the Thermotogales collection.

### DNA microarray hybridization

*T. maritima* MSB8 (ATCC:43589) was grown in 500-ml serum bottles containing 200 ml of minimal medium (Rinker and Kelly, 2000) with 10 mM of a carbon source under anoxic conditions at 80°C. Carbon sources D-glucose, D-galactose, L-rhamnose, L-arabinose, D-xylose, D-ribose, D-mannose, N-acetyl-D-glucosamine, D-gluconate, glucuronate, galacturonate, mannitol, sorbitol, trehalose, cellobiose, maltose, and pectin were obtained from Sigma (St. Louis, MO). The cell growth was monitored spectrophometrically at 600 nm. Once the mid-logarithmic phase was reached, growth was stopped with 20 ml of stop solution composed of 5% Trizol and 95% ethanol (Sigma). RNA isolation was performed using a conventional RNAeasy mini kit protocol with DNaseI treatment (Qiagen, Valencia, CA). Total RNA yields were quantified by using a NanoDrop (Thermo Fisher Scientific, Waltham, MA) at a wavelength of 260 nm, and RNA quality was checked by measuring the samples' A260/A280 ratio (>1.8). cDNA synthesis was conducted as following: Amino-allyl cDNAs were reverse transcribed from 10 μg of purified total RNA and then labeled with Cy3 monoreactive dyes (Amersham, GE HealthCare, UK). Labeled cDNA samples were fragmented to the 50–300-bp range with DNase I (EpiCentre Biotechnologies, Madison, WI) and interrogated with high-density four-plex oligonucleotide tilling arrays consisting of 4 × 71,548 probes of variable length spaced across the whole *T. maritima* genome (Roche-NimbleGen, Madison, WI). Hybridization, washing, and scanning were performed according to the manufacturer's instructions. Two biological replicates were utilized for each growth condition. Probe level data were normalized using the Robust Multiarray Analysis algorithm without background correction as implemented in NimbleScan™ 2.4 software (Roche-NimbleGen). Transcriptome data have been deposited in NCBI's Gene Expression Omnibus and are accessible through GEO Series accession number GSE47615 (http://www.ncbi.nlm.nih.gov/geo/query/acc.cgi?acc=GSE47615).

### Real time PCR

Individual transcript levels were measured for four genes from the ATCC-derived strain of *T. maritima* MSB8: *cbpA (Tmari_1862), treE (Tmari_1861), gluE (Tmari_1858)*, and *gluR (Tmari_1855)* (Genbank Accession CP004077). Genomic RNA was isolated from cells grown on minimal medium supplied with either trehalose, D-glucose, or D-ribose and collected at the same optical density using the RNeasy minikit (Qiagen, Valencia, CA). Reverse transcription was performed on 10 μg of total RNA. The reverse transcription mixture (60 μl) contained 10 μg total RNA, 75 μg random primers, 1 × 1st Strand buffer, 10 mM dithiothreitol, 0.5 mM deoxynucleoside triphosphates, 30 U Superase, and 1,500 U Superscript II. The mixture was incubated in a thermocycler (Bio-Rad, Hercules, CA) at 25°C for 10 min, 37°C for 1 h, and then 42°C for 1 h. The reaction was followed by incubation at 70°C for 10 min to inactivate the superscript. The RNA was then degraded by adding 20 μl of 1 N NaOH and incubating at 65°C for 30 min. After the incubation, 20 μl of 1 N HCl were added to neutralize the solution. QIAquick PCR purification kits were used to clean up the cDNA synthesis product. Following the purification, the cDNA was quantified and then directly used in quantitative PCR (qPCR) reactions. The 50 μl of qPCR reaction volume contained 25 μl Sybr green tag master mix (Qiagen), 0.2 μM forward primer, 0.2 μM reverse primer, and cDNA as a template. Each qPCR reaction was run in triplicate in the Bio-Rad thermocycler (Bio-Rad, Hercules, CA) with the following settings: 95°C for 15 min, 94°C for 15 s, 52°C for 30 s, and 72°C for 30 s; the denaturation, annealing, and extension steps were repeated for 40 cycles. In order to determine the binding affinity of each primer set, a standard curve was calculated for each primer and reaction efficiency obtained from it. Using the standard curve, the relative cDNA quantity was obtained for each gene by normalizing it to the quantity of *gap* (*TM0688*) cDNA in the same sample. The *gap* gene was used as an expression control as it demonstrated no differential expression across all gene expression arrays in the present study. Normalized transcript levels are provided in Figure S1 in Supplementary Material.

### Gene cloning and protein purification

The *E. coli* BL21 strains containing pMH2T7-derived plasmid that harbor the *T. maritima* TM0439 (*uxaR*) gene with the arabinose promoter and the His_6_ tag were a kind gift from Dr. S. Lesley at the Joint Center for Structural Genomics (La Jolla, CA). Recombinant UxaR protein containing an N-terminal His_6_ tag was overexpressed in *E. coli* in 50 ml volume culture and purified using Ni^2+^-chelating chromatography, as previously described (Yang et al., 2008). Briefly, cells were grown in TB medium to OD600 = 1.4 at 37°C induced by 0.15% L-arabinose and harvested after 3 h shaking at 37°C. Harvested cells were re-suspended in 20 mM HEPES buffer pH 7 containing 100 mM NaCl, 0.03% Brij 35, and 2 mM β-mercaptoethanol supplemented with 2 mM PMSF and a protease inhibitor cocktail (Sigma-Aldrich). Lysozyme was added to 1 mg/ml, and the cells were lyzed by freezing-thawing followed by sonication. After centrifugation at 18,000 rpm, the Tris-HCl buffer (pH 8) was added to the supernatant (50 mM, final concentration), and it was loaded onto an Ni-NTA acid agarose minicolumn (0.3 mL). After washing with the starting buffer containing 1 M NaCl and 0.3% Brij-35, bound proteins were eluted with 0.3 mL of the starting buffer containing 250 mM imidazole and then followed by buffer exchange. Finally, protein was suspended in 20 mM HEPES pH7.0 containing 1 mM DTT and 0.5 mM EDTA. Protein size, expression level, distribution between soluble and insoluble forms, and extent of purification were monitored by SDS PAGE. Protein concentration was determined by the Quick Start Bradford Protein Assay kit from Bio-Rad.

### DNA binding assays

The interaction of the purified recombinant UxaR protein with their cognate DNA-binding sites in *T. maritima* was assessed using the fluorescence polarization DNA-binding assay. The DNA oligos containing the predicted binding sites were synthesized by Integrated DNA Technologies. One strand of oligo was 3′-labeled by a fluorescence label, 6-carboxyfluorescin), whereas the complementary oligo was unlabeled. The double-stranded labeled DNA fragments were obtained by annealing the labeled oligonucleotides with unlabeled complementary oligonucleotides at a 1:10 ratio. The labeled 30-bp DNA fragments (1 nM) were incubated with increasing concentrations of the purified UxaR protein (10–500 nM) in a total volume of 100 μl of the binding buffer containing 20 mM Tris-HCl (pH 7.5), 100 mM NaCl, and 0.3 mg/ml BSA at 37°C for 30 min. Poly(dI-dC) was added to the reaction mixture as a non-specific competitor DNA at 1 μg to suppress non-specific binding. The fluorescence-labeled DNA was detected with the FLA-5100 fluorescent image analyzer. A DNA fragment containing the Rex regulator binding site was used as a negative control (Ravcheev et al., 2012).

### Conflict of interest statement

The authors declare that the research was conducted in the absence of any commercial or financial relationships that could be construed as a potential conflict of interest.

## Supplementary material

The Supplementary Materials for this article can be found online at: http://www.frontiersin.org/Microbial_Physiology_and_Metabolism/10.3389/fmicb.2013.00244/abstract
