# Supplementary material for: Transcriptional regulation of the carbohydrate utilization network in Thermotoga maritima
Source: Front Microbiol. 2013 Aug 23;4:244. doi: 10.3389/fmicb.2013.00244 (PMC3750489; doi:10.3389/fmicb.2013.00244)
Supplement: Supplementary file 1 [file Presentation1.PDF]

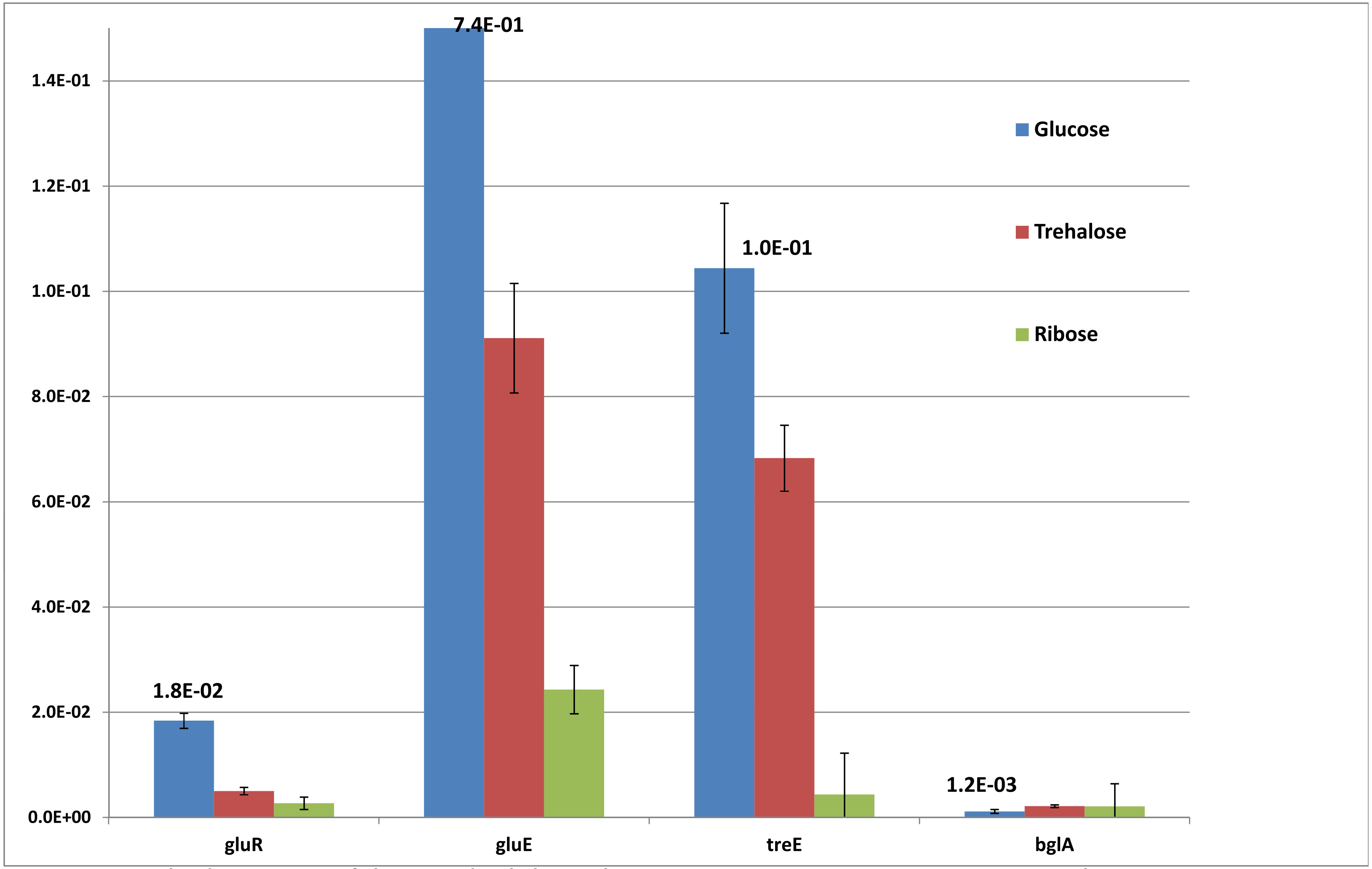

Figure S1. Normalized expression of glucose and trehalose utilization genes in *T. maritima* grown on various carbon sources. Analysis is done with respect to the TM0688 (gap) gene (used as a "house-keeping" gene). Expression of gap was not changed in the current analysis and in the entire pool. The mean of biological duplicate measurements is shown.
