# Supplementary material for: Transcriptional regulation of the carbohydrate utilization network in Thermotoga maritima
Source: Front Microbiol. 2013 Aug 23;4:244. doi: 10.3389/fmicb.2013.00244 (PMC3750489; doi:10.3389/fmicb.2013.00244)
Supplement: Supplementary file 3 [file DataSheet2.PDF]

Table S2. Distribution of carbohydrate utilization genes and their regulation in 11 Thermotogales genomes.

Path-way*	Locus tag**	Name	Function	Regulator ***	Thermotoga maritima	Thermotoga sp. RQ2	Thermotoga petrophila	Thermotoga naphthophila	Thermotoga neapolitana	Thermotoga lettingae	Thermosipho africanus	Thermosipho melanesiensis	Fervidobacterium nodosum	Petrotoga mobilis	Kosmotoga olearia	2nd regulator ****
CCM	TM0208	**pyk**	Pyruvate kinase (EC 2.7.1.40)	-	+	+	+	+	+	+	+	+	+	+	+	
CCM	TM0209	**pfk**	6-phosphofructokinase (EC 2.7.1.11)	-	+	+	+	+	+	+	+	+	+	+	+	
CCM	TM0273	**fba**	Fructose-bisphosphate aldolase class II (EC 4.1.2.13)	-	+	+	+	+	+	+	+	+	+	+	+	
CCM	TM0295	**tal**	Transaldolase (EC 2.2.1.2)	-	+	+	+	+	+	+	+	+	+	+	+	
CCM	TM0688	**gap**	NAD-dependent glyceraldehyde-3-phosphate dehydrogenase (	-	+	+	+	+	+	+	+	+	+	+	+	
CCM	TM0689	**pgk**	Phosphoglycerate kinase (EC 2.7.2.3)	-	+	+	+	+	+	+	+	+	+	+	+	
CCM	TM0689	**tpi**	Triosephosphate isomerase (EC 5.3.1.1)	-	+	+	+	+	+	+	+	+	+	+	+	
CCM	TM0877	**eno**	Enolase (EC 4.2.1.11)	-	+	+	+	+	+	+	+	+	+	+	+	
CCM	TM1080	**rpi**	Ribose 5-phosphate isomerase B (EC 5.3.1.6)	-	+	+	+	+	+	+	+	+	+	+	+	
CCM	TM1154	**pgl**	6-phosphogluconolactonase (EC 3.1.1.31), eukaryotic type	-	+	+	+	+	+					+		
CCM	TM1155	**zwf**	Glucose-6-phosphate 1-dehydrogenase (EC 1.1.1.49)	-	+	+	+	+	+					+		
CCM	TM1374	**gpm**	Phosphoglycerate mutase (EC 5.4.2.1)	-	+	+	+	+	+	+			+	+		
CCM	TM1385	**pgi**	Glucose-6-phosphate isomerase (EC 5.3.1.9)	-	+	+	+	+	+	+	+	+	+	+	+	
CCM	TM1469	**glk**	Glucokinase (EC 2.7.1.2)	-	+	+	+	+	+	+	+	+	+	+	+	
CCM	TM1585	**gckA**	D-glycerate 2-kinase (EC 2.7.1.-)	Rex	+	+	+	+	+	+	+	+	+	+	+	
CCM	TM1718	**rpe**	Ribulose-phosphate 3-epimerase (EC 5.1.3.1)	-	+	+	+	+	+	+	+	+	+	+	+	
CCM	TM1762	**tkt**	Transketolase (EC 2.2.1.1)	-	+	+	+	+	+	+	+	+	+	+	+	
CCM	TM1774	**gpm2**	2,3-bisphosphoglycerate-independent phosphoglycerate muta	-	+	+	+	+	+	+	+	+	+	+	+	
Ara	TM0275	**araR**	Transcriptional repressor of arabinoside utilization operon, Gn	AraR	+	+	+	+	+	+						
Ara	TM0276	**araA**	L-arabinose isomerase (EC 5.3.1.4)	AraR	+	+	+	+	+							
Ara	TM0277-79	**araEFG**	Predicted alpha-arabinosides ABC transporter	AraR	+	+	+	+	+							
Ara	TM0280	**TM0280**	Putative glycosyl hydrolase of unknown function (DUF1680)	AraR	+	+	+	+	+							
Ara	TM0281	**abfA**	Alpha-N-arabinofuranosidase (EC 3.2.1.55)	AraR	+	+	+	+	+	+						
Ara	TM0282	**araM**	L-arabinose-specific 1-epimerase (mutarotase)	AraR	+	+	+	+	+							
Ara	TM0283	**araD**	L-ribulose-5-phosphate 4-epimerase (EC 5.1.3.4)	AraR	+	+	+	+	+	+						
Ara	TM0284	**araB**	alternative Ribulokinase (EC 2.7.1.16)	AraR	+	+		+		+						
Ara	TM0285	**araW**	Predicted glycerol-1-phosphate dehydrogenase, arabinose ope	AraR	+			+	+							

Ara	Tlet_1149	**araI**	Predicted arabinose isomerase	AraR						+						
Ara	Tpet_0633	**abfA2**	Alpha-N-arabinofuranosidase II (EC 3.2.1.55)	AraR		+	+			+						
Ara	Tpet_0634-36	**araXYZ**	Predicted arabinoside ABC transporter II	AraR		+	+			+						
Bgl	TM0032	**bglR**	Cellobiose-responsive regulator of beta-glucosides utilization, I	BglR	+	+	+	+	+	+						
Bgl	TM0025	**bglB**	Beta-glucosidase (EC 3.2.1.21)	BglR	+	+	+	+	+	+	+	+	+	+	+	
Bgl	TM0024	**lamA**	Laminarinase (EC 3.2.1.39)	BglR	+	+	+	+	+		+		+			
Bgl	TM0026	**TM0026**	Hypothetical protein TM0026, BglR regulon	BglR	+	+	+	+	+							
Bgl	TM0027-31	**bglEFGKL**	Beta-glucoside ABC transporter	BglR	+	+	+	+	+					+		
Bgl	CTN_0660-62	**bglXYZ**	Predicted beta-glucoside-regulated ABC transporter	BglR					+	+	+	+		+	+	
Cel	TM1218	**celR**	Predicted regulator of cellobiose and glucan utilization, LacI fa	CelR	+	+	+	+	+	+	+		+			
Cel	TM1219-23	**celEFGKL**	Predicted cellobiose ABC transporter	CelR	+	+	+	+	+	+	+		+			
Cel	TM0308	**celQ**	Putative alpha-glucosidase (EC 3.2.1.-)	CelR	+	+	+	+	+							
Cel	TM0312	**TM0312**	Predicted dehydrogenase in CelR regulon, COG0673	CelR	+	+	+	+	+		+					
Cel	TM0313	**TM0313**	Predicted aldo/keto reductase in CelR regulon, COG4989	CelR	+	+	+	+	+							
Cel	TM1524	**cel12A**	Cytoplasmic endo-1,4-beta-glucanase (EC 3.2.1.4)	CelR	+	+	+	+	+							
Cel	TM1525	**cel12B**	Extracellular endo-1,4-beta-glucanase (EC 3.2.1.4)	CelR	+	+	+	+	+							
Cel	Tmari_1862	**bglA**	Beta-glucosidase A (EC 3.2.1.21)	CelR	+	+	+	+	+							
Cel	TM1848 / Tmari_	**cbpA**	Cellobiose phosphorylase (EC 2.4.1.-)	CelR	+	+	+	+	+							
Glo	TM0299	**gloR**	Predicted xyloglucan oligosaccharide utilization regulator, LacI	GloR	+	+		+		+				+		CelR
Glo	TM0300-4	**gloEFGKL**	Putative xyloglucan oligosaccharide ABC transporter	GloR	+	+										CelR
Glo	TM0305	**cel74**	Extracellular endo-1,4-glucanase	GloR	+	+										CelR
Glo	TM0306	**fucA**	Alpha-L-fucosidase (EC 3.2.1.51)	GloR	+	+	+	+	+							CelR
Glo	TM0307	**fucI**	Putative L-fucose isomerase	GloR	+	+	+	+	+	+				+		CelR
Chi	TM0808	**chiR**	Regulator of chitobiose utilization ChiR, ROK family	ChiR	+	+	+	+	+	+	+	+				
Chi	TM0809	**cbsA**	Beta-hexosaminidase (EC 3.2.1.52)	ChiR	+	+	+	+	+	+	+	+	+	+	+	
Chi	TM0810-12	**chiEFG**	Predicted chitobiose ABC transporter	ChiR	+	+	+	+	+							
Chi	TM0813	**nagB**	Glucosamine-6-phosphate deaminase [isomerizing], alternative	ChiR	+	+	+	+	+	+	+	+		+	+	
Chi	TM0814	**nagA**	N-acetylglucosamine-6-phosphate deacetylase (EC 3.5.1.25)	ChiR	+	+	+	+	+	+	+	+	+	+	+	
Chi	THA_1060-62	**chiXYZ**	Predicted chitobiose ABC transporter II	ChiR						+	+	+				
Fru	TRQ2_0642	**fruR**	Predicted regulator of fructose utilization, DeoR family	FruR		+		+								
Fru	TRQ2_0637	**ptsI**	Phosphoenolpyruvate-protein phosphotransferase of PTS syste	FruR		+		+								
Fru	TRQ2_0638	**ptsH**	Phosphotransferase system, phosphocarrier protein HPr	FruR		+		+								
Fru	TRQ2_0639-40	**fruAB**	Fructose-specific PTS transport system	FruR		+		+								
Fru	TRQ2_0641	**fruK**	1-phosphofructokinase (EC 2.7.1.56)	FruR		+		+		+	+	+	+	+	+	
Fru	TM1414	**bfrA**	Beta-fructosidase BfrA	-	+	+	+	+								
Gal	TM0509	**galE**	UDP-glucose 4-epimerase (EC 5.1.3.2)	-	+	+	+	+	+	+	+	+	+	+	+	
Gal	TM1200	**galR**	Predicted regulator of galactoside utilization, LacI family	GalR	+	+	+	+	+	+	+		+	+		
Gal	TM1190	**galK**	Galactokinase (EC 2.7.1.6)	GalR	+	+	+	+	+	+	+	+	+	+	+	

Gal	TM1191	**galT**	Galactose-1-phosphate uridylyltransferase (EC 2.7.7.10)	GalR	+	+	+	+	+	+	+	+	+	+	+
Gal	TM1192	**galA**	Alpha-galactosidase (EC 3.2.1.22)	GalR	+	+	+	+	+	+	+	+	+	+	+
Gal	TM1193	**lacZ**	Beta-galactosidase (EC 3.2.1.23), LacZ family	GalR	+	+	+	+	+	+	+				
Gal	TM1195	**lacA**	Beta-galactosidase (EC 3.2.1.23), LacA family	GalR	+	+	+	+	+				+	+	
Gal	TM1196-99	**ltpEFGKL**	Predicted galactoside ABC transporter	GalR	+	+			+						
Gal	TM1201	**ganA**	Arabinogalactan endo-1,4-beta-galactosidase (EC 3.2.1.89)	GalR	+	+	+		+	+					
Gal	TM1202-04	**ganEFG**	Galactose oligosaccharide ABC transporter	GalR	+	+	+		+	+					
Gal	CTN_1372-74	**galXYZ**	Predicted galactoside ABC transporter II	GalR					+	+			+		
Gal	Pmob_0826-28	**lacEFG**	Predicted lactose ABC transporter	GalR							+			+	
Glp	TM1120-2	**glpABC**	Glycerol-3-phosphate ABC transporter	-	+	+		+		+	+	+		+	+
Glp	TM1429	**glpF**	Glycerol uptake facilitator protein	GlpP	+										
Glp	TM1430	**glpK**	Glycerol kinase (EC 2.7.1.30)	GlpP	+	+	+	+	+	+	+	+	+	+	+
Glp	TM1431	**glpP**	Glycerol uptake operon antiterminator regulatory protein	GlpP	+	+	+	+	+	+	+	+		+	+
Glp	TM1432	**glpZ**	Glycerol-3-phosphate dehydrogenase (EC 1.1.5.3)	GlpP	+	+	+	+	+	+	+	+	+	+	+
Uxa	TM0439	**uxaR**	Regulator of pectin and galacturonate utilization, GntR family	UxaR	+	+	+	+	+						
Uxa	TM0438	**gnd**	6-phosphogluconate dehydrogenase, decarboxylating (EC 1.1.1	UxaR	+	+	+	+	+	+				+	
Uxa	TM0440	**uxaE**	D-tagaturonate epimerase	UxaR	+	+	+	+	+	+					
Uxa	TM0441	**uxuB-II**	D-mannonate dehydrogenase (EC 1.1.1.57), NADPH-dependen	UxaR	+	+	+	+	+	+					
Uxa	TM0442	**gntE**	Novel D-mannonate-D-gluconate epimerase	UxaR	+	+	+	+	+						
Uxa	TM0443	**gntK**	Gluconokinase (EC 2.7.1.12)	UxaR	+	+	+	+	+	+				+	
Uxa	TM0430-32	**aguEFG**	Alpha-1,4-digalacturonate ABC transporter	UxaR	+	+	+			+					
Uxa	TM0433	**pelA**	Pectate lyase precursor (EC 4.2.2.2)	UxaR	+	+				+					
Uxa	TM0436	**aldH**	Alcohol dehydrogenase, zinc-containing	UxaR	+	+	+			+					
Uxa	TM0437	**pelB**	Polygalacturonase (EC 3.2.1.15)	UxaR	+	+	+								
Uxa	TM0066	**kdgA**	2-dehydro-3-deoxyphosphogluconate aldolase (EC 4.1.2.14)	UxaR	+	+	+	+	+	+					
Uxa	TM0067	**kdgK**	2-dehydro-3-deoxygluconate kinase (EC 2.7.1.45)	UxaR	+	+	+	+	+	+					
Uxa	TM0068	**uxuB**	D-mannonate oxidoreductase (EC 1.1.1.57)	UxaR	+	+	+	+	+						
Uxa	TM0069	**uxuA**	Mannonate dehydratase (EC 4.2.1.8)	UxaR	+	+	+	+	+						
Uxa	CTN_0235-37	**uxaPQM**	Predicted galacturonate TRAP transporter	UxaR				+	+						
Uxa	CTN_0238-40	**uxaXYZ**	Predicted sugar ABC transporter, galacturonate-related-1	UxaR				+	+						
Uxa	Tpet_0485-89	**-**	Predicted sugar ABC transporter, galacturonate-related-2	UxaR			+								
Uxa	TRQ2_0501	**pemA**	Putative pectinesterase (EC 3.1.1.11)	UxaR		+									
Uxa	TM0435	**TM0435**	Putative methyl oligogalcturonate esterase	-	+	+									
Uxa	TM0434	**agu4A**	Alpha-glucuronidase (EC 3.2.1.139)	-	+										
Uxa	TM0752	**agu4B**	Alpha-glucuronidase (EC 3.2.1.139)	-	+	+	+	+	+						
Kdg	TM0065	**kdgR**	Predicted 2-keto-3-deoxygluconate-responsive regulator of glu	KdgR	+	+	+	+	+						
Kdg	TM0063	**TM0063**	Putative uncharacterized protein TM0063	KdgR	+	+	+	+	+						
Kdg	TM0064	**uxaC**	Uronate isomerase (EC 5.3.1.12)	KdgR	+	+	+	+	+	+				+	

Xyl	TM0055	**aguA**	Alpha-glucuronidase (EC 3.2.1.139)	XylR	+	+	+	+	+	+		+		+		KdgR
Xyl	TM0056-60	**xtpEFGKL**	Xylan oligosaccharide ABC transporter	XylR	+	+	+	+	+							KdgR
Xyl	TM0061	**xynA**	Endo-1,4-beta-xylanase A precursor (EC 3.2.1.8)	XylR	+	+	+	+	+	+				+		KdgR
Xyl	TM0062	**cenC**	Carbohydrate-binding, CenC domain protein	XylR	+		+	+								KdgR
Xyl	TM0070	**xynB**	Endo-1,4-beta-xylanase B (EC 3.2.1.8)	XylR	+	+	+	+	+							KdgR
Xyl	TM0071-5	**xloEFGKL**	Xylose oligosaccharides ABC transporter	XylR	+	+	+	+	+	+				+		KdgR
Xyl	TM0076	**xyl3**	Beta-xylosidase (EC 3.2.1.37)	XylR	+	+	+	+	+	+				+		KdgR
Xyl	TM0077	**axeA**	Acetyl xylan esterase (EC 3.1.1.41)	XylR	+	+	+	+	+	+						KdgR
Xyl	TM0110	**xylR**	Xylose-responsive transcription regulator, ROK family	XylR	+	+	+	+	+	+						
Xyl	TM0111	**adhB**	Iron-containing alcohol dehydrogenase	XylR	+	+	+	+	+	+						
Xyl	TM0113	**xylU**	Acetyl xylan esterase XylU (EC 3.1.1.41)	XylR	+	+	+	+	+							
Xyl	TM0112-15	**xylEFK**	Xylose ABC transporter	XylR	+	+	+	+	+	+						
Xyl	TM0116	**xylB**	Xylulose kinase (EC 2.7.1.17)	XylR	+	+	+	+	+	+				+		
Xyl	TM0309	**xtpN**	Predicted xylose oligosaccharide ABC transporter, substrate-bi	XylR	+	+	+	+	+							
Xyl	TM0310	**bgaL**	Beta-galactosidase (EC 3.2.1.23)	XylR	+	+	+	+	+	+						
Xyl	TM1667	**xylA**	Xylose isomerase (EC 5.3.1.5)	XylR	+	+	+	+	+					+		
Xyl	TM1668	**TM1668**	Hypothetical protein, DUF192 family	XylR	+	+	+	+	+					+		
Xyl	CTN_1302	**xynA3**	Endo-1,4-beta-xylanase C precursor (EC 3.2.1.8)	XylR		+			+							
Ino	TM0411	**iolR**	Regulator of myo-inositol utilization IolR, ROK family	IolR	+		+	+	+							
Ino	TM0412	**iolM**	Inosose dehydrogenase	IolR	+		+	+	+							
Ino	TM0413	**iolN**	Keto-inosose hydrolase	IolR	+		+	+	+							
Ino	TM0414	**iolG**	Myo-inositol 2-dehydrogenase 1 (EC 1.1.1.18)	IolR	+		+	+	+	+					+	
Ino	TM0415	**iolK**	Novel inositol-related kinase, PfkB family (EC 2.7.1.12)	IolR	+		+	+	+						+	
Ino	TM0416	**iolO**	5-keto-L-gluconate epimerase	IolR	+		+	+	+							
Ino	TM0418-21	**inoEFGK**	Myo-inositol phosphate ABC transporter	-	+		+	+	+						+	
Ino	TM0422	**inoL**	Inositol-related sugar-phosphate epimerase	-	+		+	+	+						+	
Mal	TM1834	**aglA**	Maltodextrin glucosidase (EC 3.2.1.20)	-	+	+	+	+	+	+			+		+	
Mal	TM1835	**aglB**	Neopullulanase (EC 3.2.1.135)	-	+	+	+	+	+		+	+	+	+	+	
Mal	TM1839	**malEFG**	Maltose/maltodextrin ABC transporter	-	+	+	+	+	+		+	+	+	+	+	
Mal	TM1840	**amyA**	Alpha-1,4-amylase / Alpha-1,6-pullulanase	-	+	+	+	+	+		+	+	+			
Mal	TM1841	TM1841	hypothetical carbohydrate binding protein	-	+	+	+	+	+				+			
Mal	TM1842	TM1842	hypothetical outer membrane protein	-	+	+	+	+	+				+			
Mal	TM1843	TM1843	hypothetical outer membrane protein	-	+	+	+	+	+				+			
Mal	TM1844	TM1844	glycoside hydrolase family 13 protein	-	+	+	+	+	+				+			
Mal	TM1845	**pulA**	Pullulanase (EC 3.2.1.41)	-	+	+	+	+	+	+	+	+	+	+	+	
Mal	TM1846	TM1846	Beta transducin-related protein	-	+	+	+	+	+							
Mal	TM0364	**mgtA**	4-alpha-glucanotransferase MgtA (EC 2.4.1.25)	-	+	+	+				+	+	+			
Mal	TM0767	**mmtA**	Maltodextrin glycosyltransferase MmtA	-	+	+	+			+			+			

Mal	TM1650	**amyB**	Intracellular alpha-amylase (EC 3.2.1.1)	-	+	+	+									
Man	TM1224	**manR**	Mannose-responsive regulator of mannose and mannoside uti	ManR	+	+	+	+	+							
Man	TM1225	**manC**	Predicted mannobiose phosphorylase	ManR	+	+	+	+	+	+						
Man	TM1226	**manD**	Mannoside ABC transport system, sugar-binding protein	ManR	+	+		+								
Man	TM1227	**manB**	Endo-1,4-beta-mannosidase	ManR	+	+	+	+	+							
Man	TM1746-50	**mtpEFGKL**	Beta-mannan induced ABC transporter	ManR	+	+										
Man	TM1751	**cel5A**	Endoglucanase (EC 3.2.1.4)	ManR	+	+										
Man	TM1752	**cel5B**	Endo-mannanase (EC 3.2.1.78)	ManR	+	+										
Man	TM0736	**manA**	Mannose-6-phosphate isomerase (EC 5.3.1.8)	-	+	+	+	+	+	+	+	+	+	+	+	
Man	TM1624	**man2**	Beta-mannosidase Man2	-	+	+	+							+		
Rbs	TM0949	**rbsR**	Predicted regulator of ribose utilization, LacI family	RbsR	+		+	+	+	+	+			+	+	
Rbs	TM0955-58	**rbsABC**	Ribose ABC transporter	RbsR	+		+	+	+	+	+			+	+	
Rbs	TM0959	**rbsD**	D-ribose pyranase (EC 5.5.1.n1)	RbsR	+		+	+	+		+			+	+	
Rbs	TM0960	**rbsK**	Ribokinase (EC 2.7.1.15)	RbsR	+		+	+	+	+	+			+	+	
Rbs	TM0950	**TM0950**	Hypothetical protein TM0950	RbsR	+				+							
Rbs	TM0951	**darA**	Predicted D-arabinose isomerase	RbsR	+				+	+						
Rbs	TM0952	**drlK**	Predicted D-ribulose kinase, FGGY family	RbsR	+				+	+						
Rbs	TM0953	**tktB**	Transketolase, C-terminal section (EC 2.2.1.1)	RbsR	+				+	+					+	
Rbs	TM0954	**tktA**	Transketolase, N-terminal section (EC 2.2.1.1)	RbsR	+				+	+					+	
Rbs	TM0957	**TM0957**	Hypothetical protein, no COGs	RbsR	+				+	+						
Rha	TM1069	**rhaR**	Predicted regulator of rhamnose oligosacchoride utilization, De	RhaR	+		+	+	+							
Rha	TM1061	**TM1061**	Putative unsaturated glucuronyl hydrolase	RhaR	+	+	+	+	+							
Rha	TM1062	**gusB**	Putative beta-glucuronidase	RhaR	+	+	+	+	+							
Rha	TM1063-67	**rtpEFGKL**	Predicted rhamnose oligosaccharide ABC transporter	RhaR	+	+	+	+	+							
Rha	TM1070	**rhaM**	Predicted L-rhamnose mutarotase	RhaR	+		+	+	+							
Rha	TM1071	**rhaA**	Alternative L-rhamnose isomerase (EC 5.3.1.14)	RhaR	+		+	+	+							
Rha	TM1072	**rhaD**	Rhamnulose-1-phosphate aldolase (EC 4.1.2.19)	RhaR	+	+	+	+	+							
Rha	TM1073	**rhaB**	Rhamnulokinase (EC 2.7.1.5)	RhaR	+	+	+	+	+							
Rha	TM1074	**rhaC**	Glycoside hydrolase family 2, sugar binding	RhaR	+	+	+	+	+							
Rha	TM1068	**agu4C**	Alpha-glucuronidase (EC 3.2.1.139)	RhaR	+	+	+		+							
Glu	TM1847/Tmari_1	**gluR**	Regulator of glucose and trehalose utilization, ROK family	GluR	+	+	+	+	+							
Glu	Tmari_1856-58	**gluEFK**	Glucose ABC transporter	GluR	+	+		+	+							
Tre	TM0393	**treR**	Regulator of trehalose utilization TreR, ROK family	TreR	+	+	+	+	+	+	+	+	+		+	
Tre	TM0392	**treT**	Trehalose synthase, nucleoside diphosphate glucose dependen	TreR	+	+	+	+	+	+	+	+	+		+	
Tre	Tmari_1859-61	**treEFG**	Trehalose ABC transporter	TreR	+	+	+	+	+		+	+	+	+		GluR
Tre	CTN_0781	**amyE**	Extracellular alpha-amylase precursor	TreR			+	+	+				+	+		GluR
Tre	Tlet_1842-44	**treXYZ**	Predicted trehalose ABC transporter	TreR						+					+	
Pol	TM0296	**scrK**	Fructokinase (EC 2.7.1.4)	-	+	+	+	+	+		+	+	+	+	+	

Pol	TM0297	**ardH**	D-arabitol dehydrogenase (EC 1.1.1.250)	-	+	+	+	+	+						
Pol	TM0298	**mtlD**	Thermostable mannitol dehydrogenase	-	+	+	+	+	+						
HSU	TM1228	**ugtR**	Predicted sugar catabolic transcriptional regulator UgtR, RpiR f	UgtR	+	+	+	+	+	+	+			+	+
HSU	TM1229	**TM1229**	Glycosyl transferase, family 2	UgtR	+	+	+	+	+	+				+	+
HSU	TM1230	**TM1230**	Glycosyl transferase group 1	UgtR	+	+	+	+	+	+	+	+			
HSU	TM1231	**TM1231**	Alpha-mannosidase-related protein, family 38	UgtR	+	+	+	+	+	+					
HSU	TM1232-35	**ugtEFGK**	Hypothetical sugar ABC transporter	UgtR	+	+	+	+	+	+	+	+		+	+
HSU	TM0326	**uctR**	Unknown carbohydrate utilization transcriptional regulator Uc	UctR	+	+	+	+	+						
HSU	TM0322-24	**uctMPQ**	Unknown carbohydrate transporter, TRAP family	UctR	+	+	+	+	+						
HSU	TM0325	**TM0325**	Predicted sugar dehydrogenase TM0325	UctR	+	+	+		+						
HSU	TM0327	**TM0327**	Phosphoglycerate dehydrogenase TM0327, putative	UctR	+	+	+	+	+						
HSU	TM1856	**ugpR**	Predicted regulator of alpha-mannoside utilization, LacI family	UgpR	+		+	+	+	+	+	+			
HSU	TM1851	**mnnA**	Alpha-mannosidase (EC 3.2.1.24)	UgpR	+	+	+	+	+	+	+	+	+	+	+
HSU	TM1852	**TM1852**	Predicted glycosylase, COG2152	UgpR	+		+	+	+	+	+	+			
HSU	TM1853-55	**ugpEFG**	Predicted alpha-mannoside ABC transporter	UgpR	+		+	+	+	+	+				
HSU	TM0795	**TM0795**	Hypothetical sugar kinase TM0795, PfkB family	-	+		+	+	+	+				+	+
HSU	TM0828	**namK**	Novel N-acetylmannosamine kinase	-	+	+	+	+	+	+	+	+	+	+	+
HSU	TM1280	**bglK**	Novel glucosamine kinase, PF00869 family	-	+	+	+	+	+	+					
HSU	TM1281	**bglT**	6-phospho-beta-glucosidase BglT (EC 3. 2.1.86)	-	+	+	+	+	+	+					

* Abbreviations for Sugar utilization pathways are listed in Table 1; CCM, central carbohydrate metabolism; HSU, hypothetical sugar utilization.

** Genes coding for regulators and transporters are shown in red and blue, respectively.

*** Genes predicted to be a part of a specific sugar-responsive regulon are highlighted by a respective color.

**** Genes regulated by two different regulators are shown by red '+'.
