# Supplementary material for: Transcriptional regulation of the carbohydrate utilization network in Thermotoga maritima
Source: Front Microbiol. 2013 Aug 23;4:244. doi: 10.3389/fmicb.2013.00244 (PMC3750489; doi:10.3389/fmicb.2013.00244)
Supplement: Supplementary file 4 [file DataSheet3.PDF]

Table S3. Differential expression of genes from reconstructed regulons in *T.maritima* grown on simple sugars and pectin.

Gene ID	Gene name	Ara / Rib	Xyl / Rib	Tre / Rib	Rha / Rib	Glu / Rib	Gal / Rib	Cell / Rib	Pectin/ Rib	Rib / Glu	Xyl / Glu	Regulons	Function
TM0024	lamA	-3	-1	1	1	11	1	5	4	-11	-12	BglR	Laminarinase (EC 3.2.1.39)
TM0025	bglB	-3	1	2	1	8	-1	6	4	-8	-7	BglR	Beta-glucosidase (EC 3.2.1.21)
TM0026	TM0026	-1	-1	2	1	9	1	6	5	-9	-10	BglR	Hypothetical protein TM0026, BglR regulon
TM0027	bglL	-2	1	1	1	7	-1	5	5	-7	-6	BglR	Beta-glucoside ABC transport system, ATP-binding protein 2
TM0028	bglK	-3	-1	1	1	6	-1	5	4	-6	-7	BglR	Beta-glucoside ABC transport system, ATP-binding protein 1
TM0029	bglG	-2	1	2	1	8	1	7	5	-8	-8	BglR	Beta-glucoside ABC transport system, permease protein 2
TM0030	bglF	-2	-1	1	1	8	-1	5	5	-8	-9	BglR	Beta-glucoside ABC transport system, permease protein 1
TM0031	bglE	-2	1	2	2	9	2	5	6	-9	-8	BglR	Beta-glucoside ABC transport system, sugar-binding protein
TM0032	bglR	-2	3	1	-1	3	-1	5	1	-3	-1	BglR	Cellobiose-responsive regulator of beta-glucosides utilization, ROK family
TM0055	aguA	-2	8	-2	1	-1	-1	-1	-3	1	9	XylR, KdgR	Alpha-glucuronidase (EC 3.2.1.139)
TM0056	xtpE	-1	8	-2	2	2	1	-2	-3	-2	4	XylR, KdgR	Xylan oligosaccharide ABC transporter, substrate-binding component
TM0057	xtpL	-2	11	-2	1	1	-2	-2	-3	-1	11	XylR, KdgR	Xylan oligosaccharide ABC transporter, ATP-binding protein 2
TM0058	xtpK	-2	15	-2	1	1	-1	-1	-3	-1	15	XylR, KdgR	Xylan oligosaccharide ABC transporter, ATP-binding protein 1
TM0059	xtpG	-2	13	-2	1	1	-1	-1	-3	-1	12	XylR, KdgR	Xylan oligosaccharide ABC transporter, permease component 2
TM0060	xtpF	-1	10	-3	1	-1	-2	-2	-4	1	13	XylR, KdgR	Xylan oligosaccharide ABC transporter, permease component 1
TM0061	xynA	-1	12	-2	1	-1	-1	-2	-4	1	16	XylR, KdgR	Endo-1,4-beta-xylanase A precursor (EC 3.2.1.8)
TM0062	cenC	-2	10	-3	1	-2	-2	-2	-4	2	15	XylR, KdgR	Carbohydrate-binding, CenC domain protein
TM0063	TM0063	1	-1	-1	-2	1	-1	-1	-2	-1	-1	KdgR	Putative uncharacterized protein TM0063
TM0064	uxaC	1	-1	-1	-2	-1	-1	-1	-2	1	1	KdgR	Uronate isomerase (EC 5.3.1.12)
TM0065	kdgR	-1	-2	-1	-4	-1	-3	-2	-1	1	-2	KdgR	Predicted 2-keto-3-deoxygluconate-responsive regulator of glucuronate utilization, IclR family
TM0066	kdgA	-1	-1	1	-4	-2	-2	-1	3	2	1	UxaR	2-dehydro-3-deoxyphosphogluconate aldolase (EC 4.1.2.14)
TM0067	kdgK	-1	-1	-1	-3	-2	-2	-1	3	2	1	UxaR	2-dehydro-3-deoxygluconate kinase (EC 2.7.1.45)
TM0068	uxuB	-1	-1	1	-3	-2	-2	-1	3	2	1	UxaR	D-mannonate oxidoreductase (EC 1.1.1.57)
TM0069	uxuA	-2	-1	-1	-3	-2	-3	-1	2	2	1	UxaR	Mannonate dehydratase (EC 4.2.1.8)
TM0070	xynB	3	8	-1	5	2	2	1	-5	-2	4	XylR, KdgR	Endo-1,4-beta-xylanase B (EC 3.2.1.8)
TM0071	xloE	4	26	-3	5	2	1	-1	-7	-2	11	XylR, KdgR	Xylose oligosaccharides ABC transporter, sugar-binding protein
TM0072	xloF	2	9	-1	2	2	1	-1	-2	-2	6	XylR, KdgR	Xylose oligosaccharides ABC transporter, permease protein 1
TM0073	xloG	1	9	-1	2	1	1	-1	-3	-1	7	XylR, KdgR	Xylose oligosaccharides ABC transporter, permease protein 2
TM0074	xloK	-1	8	-1	1	1	-1	-1	-3	-1	7	XylR, KdgR	Xylose oligosaccharides ABC transporter, ATP-binding protein 1
TM0075	xloL	2	15	2	3	2	2	2	-1	-2	7	XylR, KdgR	Xylose oligosaccharides ABC transporter, ATP-binding protein 2
TM0076	xyl3	2	15	2	3	2	2	3	1	-2	6	XylR, KdgR	Beta-xylosidase (EC 3.2.1.37)
TM0077	axeA	-1	12	1	4	2	1	2	-3	-2	5	XylR, KdgR	Acetyl xylan esterase (EC 3.1.1.41)
TM0110	xylR	1	6	-1	-1	-1	-1	-1	-3	1	8	XylR	Xylose-responsive transcription regulator, ROK family
TM0111	adhB	-1	2	-1	1	-1	-1	-2	-1	1	3	XylR	Iron-containing alcohol dehydrogenase
TM0112	xylF	1	3	1	2	1	1	-1	-2	-1	2	XylR	Xylose ABC transporter, permease component
TM0113	xylU	-2	2	-1	2	-1	-1	-2	-2	1	2	XylR	Acetyl xylan esterase XylU (EC 3.1.1.41)
TM0114	xylE	-2	1	-1	2	2	2	-2	1	-2	-1	XylR	Xylose ABC transporter, substrate-binding component
TM0115	xylK	-1	2	-1	1	1	1	1	-2	-1	2	XylR	Xylose ABC transporter, ATP-binding component
TM0116	xylB	-1	4	-1	1	-1	1	1	-2	1	4	XylR	Xylulose kinase (EC 2.7.1.17)
TM0276	araA	55	-1	-2	1	-1	-1	-1	6	1	-1	AraR	L-arabinose isomerase (EC 5.3.1.4)
TM0277	araE	13	-1	-2	3	2	1	-1	4	-2	-2	AraR	Predicted alpha-arabinosides ABC transport system, substrate-binding protein
TM0278	araF	7	-1	-1	2	1	1	-1	2	-1	-1	AraR	Predicted alpha-arabinosides ABC transport system, permease protein 1
TM0279	araG	7	-1	-1	2	1	1	1	1	-1	-1	AraR	Predicted alpha-arabinosides ABC transport system, permease protein 2
TM0280	TM0280	143	3	-1	3	2	2	1	4	-2	2	AraR	Putative glycosyl hydrolase of unknown function (DUF1680)
TM0281	abfA	89	2	-2	2	2	2	-1	7	-2	1	AraR	Alpha-N-arabinofuranosidase (EC 3.2.1.55)
TM0282	araM	2	-2	-3	-2	-1	1	-2	-2	1	-1	AraR	L-arabinose-specific 1-epimerase (mutarotase)
TM0283	araD	6	-1	-1	-1	-1	1	-1	2	1	-1	AraR	L-ribulose-5-phosphate 4-epimerase (EC 5.1.3.4)
TM0284	araB	4	-1	-1	-1	-1	-1	-1	2	1	1	AraR	alternative Ribulokinase (EC 2.7.1.16)
TM0285	araW	3	-1	-2	-1	-1	-1	-1	-1	1	1	AraR	Predicted glycerol-1-phosphate dehydrogenase, arabinose operon
TM0299	gloR	-2	-1	-2	-1	-1	-2	-1	-2	1	-1	GloR, CelR	Predicted xyloglucan oligosaccharide utilization regulator, LacI family
TM0300	gloE	-2	-1	-2	2	1	3	2	2	-1	-1	GloR, CelR	Putative xyloglucan oligosaccharide ABC transport system, sugar-binding protein
TM0301	gloF	-1	-1	-2	1	-1	1	-1	-1	1	-1	GloR, CelR	Putative xyloglucan oligosaccharide ABC transport system, permease protein 1
TM0302	gloG	-1	-1	-1	1	1	1	1	-1	-1	-1	GloR, CelR	Putative xyloglucan oligosaccharide ABC transport system, permease protein 2
TM0303	gloK	-2	-1	-2	1	-1	1	-1	-2	1	-1	GloR, CelR	Putative xyloglucan oligosaccharide ABC transport system, ATP-binding protein 1
TM0304	gloL	-2	-1	-2	1	-1	-1	-1	-2	1	-1	GloR, CelR	Putative xyloglucan oligosaccharide ABC transport system, ATP-binding protein 2
TM0305	cel74	-2	-2	-2	1	1	-1	-1	-2	-1	-2	GloR, CelR	Extracellular endo-1,4-glucanase
TM0306	fucA	-2	-1	-2	1	1	-1	1	-3	-1	-1	GloR, CelR	Alpha-L-fucosidase (EC 3.2.1.51)
TM0307	fucI	1	-1	-2	1	-1	2	-1	-6	1	-1	GloR, CelR	Putative L-fucose isomerase
TM0308	celQ	-1	1	-1	-1	1	-1	12	-3	-1	1	CelR	Putative alpha-glucosidase (EC 3.2.1.-)

Gene ID	Gene name	Ara / Rib	Xyl / Rib	Tre / Rib	Rha / Rib	Glu / Rib	Gal / Rib	Cell / Rib	Pectin/ Rib	Rib / Glu	Xyl / Glu	Regulons	Function
TM0309	xtpN	1	5	-3	-1	1	-1	-2	-4	-1	4	XylR	Predicted xylose oligosaccharide ABC transporter, substrate-binding component
TM0310	bgaL	-1	6	-3	-1	-1	-2	-2	-4	1	9	XylR	Beta-galactosidase (EC 3.2.1.23)
TM0312	TM0312	-1	1	-1	2	2	2	7	-2	-2	-2	CelR	Predicted dehydrogenase in CelR regulon, COG0673
TM0313	TM0313	-1	1	1	1	1	1	5	1	-1	-1	CelR	Predicted aldo/keto reductase in CelR regulon, COG4989
TM0322	uctP	2	-2	-3	1	-2	-2	1	-10	2	-1	UctR	Unknown carbohydrate transporter from TRAP family, substrate-binding component UctP
TM0323	uctM	1	-2	-2	1	-1	-2	-1	-3	1	-1	UctR	Unknown carbohydrate transporter from TRAP family, small transmembrane component UctM
TM0324	uctQ	1	-2	-2	1	-1	-2	1	-3	1	-1	UctR	Unknown carbohydrate transporter from TRAP family, large transmembrane component UctQ
TM0325	TM0325	-1	-2	-2	1	-1	-1	1	-3	1	-1	UctR	Predicted sugar dehydrogenase TM0325
TM0326	uctR	1	-1	-1	1	-1	-1	1	-4	1	1	UctR	Unknown carbohydrate utilization transcriptional regulator UctR, RpiR family
TM0327	TM0327	-1	-1	-2	1	-1	-1	1	-3	1	-1	UctR	Phosphoglycerate dehydrogenase TM0327, putative
TM0392	treT	-2	-1	62	-1	4	-2	2	2	-4	-6	TreR	Trehalose synthase, nucleoside diphosphate glucose dependent
TM0393	treR	-2	-1	13	1	2	1	1	-2	-2	-2	TreR	Regulator of trehalose utilization TreR, ROK family
TM0411	iolR	1	1	-1	1	1	1	1	-1	-1	-1	IolR	Regulator of myo-inositol utilization IolR, ROK family
TM0412	iolM	-1	-1	-2	1	2	1	-1	-2	-2	-2	IolR	Inosose dehydrogenase
TM0413	iolN	-1	-1	-2	1	2	2	1	-3	-2	-2	IolR	Keto-inosose hydrolase
TM0414	iolG	-1	1	-1	2	2	1	1	-2	-2	-1	IolR	Myo-inositol 2-dehydrogenase 1 (EC 1.1.1.18)
TM0415	iolK	-1	1	-1	2	2	1	1	-2	-2	-1	IolR	Novel inositol-related kinase, PfkB family (EC 2.7.1.12)
TM0416	iolO	-1	1	-1	2	1	1	2	-2	-1	-1	IolR	5-keto-L-gluconate epimerase
TM0430	aguG	-2	-1	-1	-1	1	2	-1	21	-1	-1	UxaR	Alpha-1,4-digalacturonate ABC transporter, permease protein 2
TM0431	aguF	-1	-1	1	-1	1	2	1	28	-1	-2	UxaR	Alpha-1,4-digalacturonate ABC transporter, permease protein 1
TM0432	aguE	-1	1	2	1	2	3	-1	24	-2	-2	UxaR	Alpha-1,4-digalacturonate ABC transporter, substrate-binding protein
TM0433	pelA	1	2	-1	2	2	3	1	79	-2	-1	UxaR	Pectate lyase precursor (EC 4.2.2.2)
TM0436	aldH	1	1	-2	-1	-1	-2	-1	8	1	1	UxaR	Alcohol dehydrogenase, zinc-containing
TM0437	pelB	1	1	-2	1	-1	-2	-1	12	1	2	UxaR	Polygalacturonase (EC 3.2.1.15)
TM0438	gnd	-1	1	1	1	-1	-1	1	4	1	2	UxaR	6-phosphogluconate dehydrogenase, decarboxylating (EC 1.1.1.44)
TM0439	uxaR	1	1	-1	-1	1	1	-1	2	-1	-1	UxaR	Regulator of pectin and galacturonate utilization, GntR family
TM0440	uxaE	-1	2	1	-1	-1	1	1	4	1	2	UxaR	D-tagaturonate epimerase
TM0441	uxuB-II	-1	2	1	1	-1	-1	1	3	1	2	UxaR	D-mannonate dehydrogenase (EC 1.1.1.57), NADPH-dependent
TM0442	gntE	-1	2	1	-1	-1	-1	1	2	1	2	UxaR	Novel D-mannonate-D-gluconate epimerase
TM0443	gntK	-1	2	-1	-1	-1	1	1	2	1	2	UxaR	Gluconokinase (EC 2.7.1.12)
TM0808	chiR	-2	-1	-2	1	1	-2	1	-2	-1	-2	ChiR	Regulator of chitobiose utilization ChiR, ROK family
TM0809	cbsA	-2	-1	-2	1	1	-1	1	-2	-1	-2	ChiR	Beta-hexosaminidase (EC 3.2.1.52)
TM0810	chiE	-2	-2	-1	-1	1	1	1	-2	-1	-3	ChiR	Predicted chitobiose ABC transport system, sugar-binding protein
TM0811	chiF	-2	-2	-2	-1	-1	-1	-1	-2	1	-2	ChiR	Predicted chitobiose ABC transport system, permease protein 1
TM0812	chiG	-2	-2	-2	-1	-1	-1	-1	-2	1	-2	ChiR	Predicted chitobiose ABC transport system, ATPase component
TM0813	nagB	-2	-1	-2	-1	-1	-1	1	-2	1	-1	ChiR	Glucosamine-6-phosphate deaminase [isomerizing], alternative (EC 3.5.99.6)
TM0814	nagA	-2	-1	-2	1	1	-1	1	-3	-1	-1	ChiR	N-acetylglucosamine-6-phosphate deacetylase (EC 3.5.1.25)
TM0949	rbsR	-5	-1	-4	-5	-4	-4	-6	-14	4	3	RbsR	Predicted regulator of ribose utilization, LacI family
TM0950	TM0950	-66	-1	-36	-25	-36	-46	-34	-112	36	35	RbsR	Hypothetical protein TM0950
TM0951	darA	-42	1	-27	-31	-35	-31	-26	-100	35	38	RbsR	Predicted D-arabinose isomerase
TM0952	drlK	-53	1	-44	-32	-35	-44	-32	-113	35	39	RbsR	Predicted D-ribulose kinase, FGGY family
TM0953	tktB	-57	1	-63	-39	-43	-47	-37	-169	43	45	RbsR	Transketolase, C-terminal section (EC 2.2.1.1)
TM0954	tktA	-42	-1	-65	-43	-40	-36	-41	-197	40	38	RbsR	Transketolase, N-terminal section (EC 2.2.1.1)
TM0955	rbsC	-81	-1	-91	-46	-39	-45	-57	-138	39	34	RbsR	Ribose ABC transport system, permease protein RbsC (TC 3.A.1.2.1)
TM0956	rbsA	-95	-1	-99	-49	-52	-54	-83	-134	52	42	RbsR	Ribose ABC transport system, ATP-binding protein RbsA (TC 3.A.1.2.1)
TM0957	TM0957	-112	-1	-156	-74	-69	-62	-118	-214	69	56	RbsR	Hypothetical protein, no COGs
TM0958	rbsB	-22	1	-42	-20	-18	-12	-51	-136	18	18	RbsR	Ribose ABC transport system, periplasmic ribose-binding protein RbsB (TC 3.A.1.2.1)
TM0959	rbsD	-85	1	-126	-68	-62	-75	-104	-98	62	63	RbsR	D-ribose pyranase (EC 5.5.1.n1)
TM0960	rbsK	-110	-1	-126	-75	-77	-82	-90	-138	77	66	RbsR	Ribokinase (EC 2.7.1.15)
TM1061	TM1061	-2	-1	-2	4	-1	-1	-1	-3	1	-1	RhaR	Putative unsaturated glucuronyl hydrolase
TM1062	gusB	-2	-1	-2	4	-1	-1	-1	-4	1	1	RhaR	Putative beta-glucuronidase
TM1063	rtpL	-2	1	-2	7	-1	-2	1	-3	1	1	RhaR	Predicted rhamnose oligosaccharide ABC transporter, ATP-binding component 1
TM1064	rtpK	-2	1	-2	11	-1	-1	1	-2	1	1	RhaR	Predicted rhamnose oligosaccharide ABC transporter, ATP-binding component 2
TM1065	rtpG	-2	-1	-2	13	-1	-1	-1	-2	1	-1	RhaR	Predicted rhamnose oligosaccharide ABC transporter, permease component 2
TM1066	rtpF	-2	-1	-2	18	-1	-2	-1	-2	1	-1	RhaR	Predicted rhamnose oligosaccharide ABC transporter, permease component 1

Gene ID	Gene name	Ara / Rib	Xyl / Rib	Tre / Rib	Rha / Rib	Glu / Rib	Gal / Rib	Cell / Rib	Pectin/ Rib	Rib / Glu	Xyl / Glu	Regulons	Function
TM1067	rtpE	-2	-2	-2	56	1	1	-1	-2	-1	-2	RhaR	Predicted rhamnose oligosaccharide ABC transporter, substrate-binding component
TM1068	agu4C	-1	-2	1	14	-1	-1	-1	-1	1	-1	RhaR	Alpha-glucuronidase (EC 3.2.1.139)
TM1069	rhaR	1	-2	1	15	-1	1	-1	-1	1	-1	RhaR	Predicted regulator of rhamnose oligosacchoride utilization, DeoR family
TM1070	rhaM	-2	-2	-1	35	-1	-1	-1	-3	1	-1	RhaR	Predicted L-rhamnose mutarotase
TM1071	rhaA	-2	-2	-1	32	-1	-1	1	-4	1	-1	RhaR	Alternative L-rhamnose isomerase (EC 5.3.1.14)
TM1072	rhaD	-1	1	-1	29	-1	-1	1	-3	1	1	RhaR	Rhamnulose-1-phosphate aldolase (EC 4.1.2.19)
TM1073	rhaB	-2	2	-1	30	-1	-1	2	-3	1	2	RhaR	Rhamnulokinase (EC 2.7.1.5)
TM1074	rhaC	-2	1	-1	24	-1	-1	2	-3	1	1	RhaR	Glycoside hydrolase family 2, sugar binding
TM1190	galK	-3	-2	-2	-1	-1	3	-1	4	1	-1	GalR	Galactokinase (EC 2.7.1.6)
TM1191	galT	-3	-1	-2	-1	-1	4	-1	6	1	1	GalR	Galactose-1-phosphate uridylyltransferase (EC 2.7.7.10)
TM1192	galA	-2	1	-1	-1	-1	5	-1	7	1	1	GalR	Alpha-galactosidase (EC 3.2.1.22)
TM1193	lacZ	-3	1	-2	-1	-1	4	-1	3	1	2	GalR	Beta-galactosidase (EC 3.2.1.23), LacZ family
TM1194	ltpL	-2	1	-2	-1	-1	6	-1	5	1	2	GalR	Predicted galactoside ABC transporter, ATP-binding protein 2
TM1195	lacA	-2	1	-2	-1	-2	8	-1	7	2	2	GalR	Beta-galactosidase (EC 3.2.1.23), LacA family
TM1196	ltpK	-3	1	-3	-1	-2	7	-2	4	2	2	GalR	Predicted galactoside ABC transporter, ATP-binding protein 1
TM1197	ltpG	-2	-1	-2	1	-1	14	-2	7	1	-1	GalR	Predicted galactoside ABC transporter, permease protein 2
TM1198	ltpF	-1	-1	-2	1	-1	19	-2	12	1	1	GalR	Predicted galactoside ABC transporter, permease protein 1
TM1199	ltpE	-2	-1	-1	1	-1	19	-3	15	1	-1	GalR	Predicted galactoside ABC transporter, sugar-binding protein
TM1200	galR	-3	-1	-2	1	-2	4	-1	1	2	1	GalR	Predicted regulator of galactoside utilization, LacI family
TM1201	ganA	-2	-1	-2	1	1	10	-1	4	-1	-1	GalR	Arabinogalactan endo-1,4-beta-galactosidase (EC 3.2.1.89)
TM1202	ganG	-1	-1	-2	1	-1	12	-1	4	1	-1	GalR	Galactose oligosaccharide ABC transporter, permease protein 2
TM1203	ganF	-2	-2	-2	1	-1	10	-2	5	1	-2	GalR	Galactose oligosaccharide ABC transporter, permease protein 1
TM1204	ganE	-1	-1	-2	1	1	5	-2	3	-1	-1	GalR	Galactose oligosaccharide ABC transporter, substrate binding protein
TM1218	celR	-2	1	-1	2	-1	-1	6	-3	1	2	CelR	Predicted regulator of cellobiose and glucan utilization, LacI family
TM1219	celL	-1	1	-1	1	-1	-1	5	-2	1	1	CelR	Predicted cellobiose ABC transport system, ATP-binding protein 2
TM1220	celK	-1	-1	1	1	1	1	6	-1	-1	-1	CelR	Predicted cellobiose ABC transport system, ATP-binding protein 1
TM1221	celG	1	-1	1	1	1	1	8	1	-1	-2	CelR	Predicted cellobiose ABC transport system, permease protein 2
TM1222	celF	1	-2	-1	1	1	1	6	1	-1	-3	CelR	Predicted cellobiose ABC transport system, permease protein 1
TM1223	celE	-1	-2	-1	2	1	2	9	1	-1	-3	CelR	Predicted cellobiose ABC transport system, sugar-binding protein
TM1224	manR	-2	-1	-2	2	1	-1	1	-4	-1	-1	ManR	Mannose-responsive regulator of mannose and mannoside utilization, ROK family
TM1225	manC	-2	-1	-2	1	1	-1	1	-3	-1	-1	ManR	Predicted mannobiose phosphorylase
TM1226	manD	-2	-1	-2	1	1	1	2	-2	-1	-2	ManR	Mannoside ABC transport system, sugar-binding protein
TM1227	manB	-2	-1	-1	1	1	-1	1	-2	-1	-2	ManR	Endo-1,4-beta-mannosidase
TM1228	ugtR	1	-1	1	1	-1	1	1	-4	1	1	UgtR	Predicted sugar catabolic transcriptional regulator UgtR, RpiR family
TM1229	TM1229	-1	-1	-1	1	1	1	1	-2	-1	-2	UgtR	Glycosyl transferase, family 2
TM1230	TM1230	-1	-1	-1	1	2	2	1	-1	-2	-2	UgtR	Glycosyl transferase group 1
TM1231	TM1231	-1	1	-1	2	2	2	1	-1	-2	-1	UgtR	Alpha-mannosidase-related protein, family 38
TM1232	ugtK	-1	-1	-1	2	2	2	1	-1	-2	-2	UgtR	Hypothetical sugar ABC transporter, ATP-binding UgtK
TM1233	ugtG	-1	-1	-1	2	2	3	1	-1	-2	-2	UgtR	Hypothetical sugar ABC transporter, membrane protein UgtG
TM1234	ugtF	-1	-1	-1	2	2	3	1	-1	-2	-3	UgtR	Hypothetical sugar ABC transporter, membrane protein UgtF
TM1235	ugtE	-2	-1	-1	3	4	6	1	-1	-4	-6	UgtR	Hypothetical sugar ABC transporter, solute-binding protein UgtE
TM1524	cel12A	-1	-1	-2	-1	-1	-3	6	-4	1	1	CelR	Cytoplasmic endo-1,4-beta-glucanase (EC 3.2.1.4)
TM1525	cel12B	2	-1	-2	1	-1	-3	6	-3	1	1	CelR	Extracellular endo-1,4-beta-glucanase (EC 3.2.1.4)
TM1667	xylA	1	13	-2	-1	-2	-2	-1	-5	2	21	XylR	Xylose isomerase (EC 5.3.1.5)
TM1668	TM1668	-1	3	-2	1	-1	-1	-1	-4	1	4	XylR	Hypothetical protein, DUF192 family
TM1746	mtpE	-2	-2	-2	2	2	-1	1	1	-2	-5	ManR	Beta-mannan induced hypothetical ABC transporter, periplasmic oligopeptide-binding protein
TM1747	mtpF	-2	-2	-2	2	1	-1	-1	1	-1	-2	ManR	Beta-mannan nduced hypothetical ABC transporter, permease protein 1
TM1748	mtpG	-2	-1	-2	1	1	1	-1	-1	-1	-2	ManR	Beta-mannan induced hypothetical ABC transporter, permease protein 2
TM1749	mtpK	-2	-1	-2	1	-1	-1	-1	-1	1	-1	ManR	Beta-mannan induced hypothetical ABC transporter, ATP-binding protein 1
TM1750	mtpL	-3	-2	-2	-1	-1	-1	-2	1	1	-1	ManR	Beta-mannan induced hypothetical ABC transporter, ATP-binding protein 2
TM1751	cel5A	-2	-1	-2	-1	1	-1	-2	1	-1	-1	ManR	Endoglucanase (EC 3.2.1.4)
TM1752	cel5B	-3	-1	-3	-1	-1	-1	-2	-1	1	-1	ManR	Endo-mannanase (EC 3.2.1.78)
TM1847	gluR	1	2	1	2	4	4	1	-2	-4	-3	GluR	Regulator of glucose and trehalose utilization, ROK family
TM1848	cbpA	3	2	-1	-1	1	-1	17	-2	-1	2	CelR	Cellobiose phosphorylase (EC 2.4.1.-)
TM1851	mnnA	-1	2	1	1	-1	-1	1	-4	1	3	UgpR	Alpha-mannosidase (EC 3.2.1.24)
TM1852	TM1852	1	1	1	-1	-2	-2	-1	-2	2	2	UgpR	Predicted glycosylase, COG2152
TM1853	ugpG	-1	-1	-2	2	1	2	-1	-3	-1	-2	UgpR	Predicted alpha-mannoside ABC transporter, permease protein 2
TM1854	ugpF	-1	-1	-3	3	1	2	-1	-3	-1	-2	UgpR	Predicted alpha-mannoside ABC transporter, permease protein 1
TM1855	ugpE	-1	-1	-2	2	-1	2	-2	-2	1	-1	UgpR	Predicted alpha-mannoside ABC transporter, substrate binding protein
TM1856	ugpR	-1	2	-2	1	-1	1	1	-3	1	3	UgpR	Predicted regulator of alpha-mannoside utilization, LacI family
